# Liquid Storage and Cryopreservation of Ram Semen: Storage-Associated Damage and the Role of Non-Enzymatic Antioxidants

**DOI:** 10.3390/vetsci13080818

**Published:** 2026-08-17

**Authors:** Tariq Sohail, Mohamed Tharwat, Aftab Shaukat, Nourhan Nassar, Fuhao Chen, Xiaomei Sun, Fahad A. Alshanbari, Yongjun Li

**Affiliations:** 1 Key Laboratory for Animal Genetics & Molecular Breeding of Jiangsu Province, College of Animal Science and Technology, Yangzhou University, Yangzhou 225009, China; drtariqsohail34@yahoo.com (T.S.);; 2 Department of Clinical Sciences, College of Veterinary Medicine, Qassim University, P.O. Box 6622, Buraidah 51452, Saudi Arabia; atieh@qu.edu.sa; 3 Animal Science Division, Department of Modern Agriculture, College of Life and Environmental Sciences, Hunan University of Arts and Science, Changde 415000, China; 4 Department of Clinical Pathology, Faculty of Veterinary Medicine, Benha University, Moshtohor 13736, Egypt; 5 Department of Medical Biosciences, College of Veterinary Medicine, Qassim University, P.O. Box 6622, Buraidah 51452, Saudi Arabia

**Keywords:** ram semen, oxidative stress, reactive oxygen species, non-enzymatic antioxidants, cryopreservation, semen extenders

## Abstract

The successful use of Assisted Reproductive Technologies (ARTs) such as artificial insemination (AI) in small ruminants and other mammals depends on many factors, the most important of which is the quality of the semen used. The liquid storage and cryopreservation of spermatozoa at lower temperatures are associated with artificial insemination and rapid genetic improvement programs in the sheep production industry. Several studies have reported increased lipid peroxidation (LPO) and highly reactive oxygen species (ROS) production during liquid storage or cryopreservation of ram semen, leading to oxidative stress (OS), decreased antioxidant defense, and changes in sperm quality parameters like biokinetic and biochemical characteristics, viability, functional membrane and DNA integrity, along with mitochondrial activity. Therefore, supplementing ram semen extenders with exogenous antioxidants before preservation could mitigate this harmful effect. Various in vitro studies have reported improvements in ram sperm quality parameters like motility indexes, vitality, functional membrane/DNA integrity, antioxidant enzyme activity, total antioxidant content, mitochondrial activity, in vivo/in vitro fertility with significant decline in sperm abnormality, free radical production, LPO, ROS, apoptosis rate, and cytochrome C release from the mitochondrial matrix after the addition of various natural and synthetic antioxidant (vitamins, glutathione, taurine, pyruvate, melatonin, cysteine, selenium, zinc, plant extracts, sugars, amino acids, polyphenols) substances during preservation. Therefore, this review summarizes recent findings on oxidative stress-induced damage to sperm quality parameters in various ram breeds during chilling storage and cryopreservation. Moreover, supplementing basic semen extenders with different non-enzymatic antioxidant substances as a method to maintain sperm quality—along with their efficacy in reducing or preventing sperm damage during preservation—was discussed in detail.

## Simple Summary

Maintaining sheep semen quality during liquid storage or cryopreservation remains a major challenge in the sheep production industry, especially during artificial insemination programs. Ram spermatozoa are highly susceptible to oxidative stress damage during the preservation process due to abrupt temperature fluctuation, which reduces their biological, morphological, biochemical, functional, and fertility-related quality parameters. This study investigated the damages that various ram breed sperm undergo during the liquid storage or cryopreservation process. Moreover, this review addresses the supplementation of different classes of non-enzymatic antioxidants into basic extenders as cryoprotective agents. Their efficiency in reducing or preventing ram spermatozoa damage during liquid storage and cryopreservation is also discussed.

## 1. Introduction

In small ruminant production, sheep farming has an advantage because ovines are highly adaptive to an open grazing system with a high conversion ratio of fibrous, low-quality feedstuffs into meat, milk, wool, and other valuable products compared to large ruminants. In the modern age, the optimization of reproduction in small ruminants is attained through reproductive biotechnology and assisted reproductive techniques, which include semen collection, preservation, the utilization of estrus synchronization techniques with artificial insemination, and in vitro embryo production technology [1]. The outcomes of these diverse practices mostly depend upon sperm quality, which includes semen collection, initial evaluation, processing, and the supplementation of the extenders to sustain sperm ultra-structural, biochemical, and functional features during preservation [2]. Semen-preservation methods, which involved either liquid storage at low temperature or cryopreservation at −196 °C, were used to reduce the metabolic rate of sperm to prolong their functional lifetime [3]. However, sperm quality indices and fertilization ability are reduced as the semen-preservation time increases. It has been documented that semen preservation procedures cause an overproduction of reactive oxygen species, including free radicals (superoxide, hydroxyl radical, peroxyl radical, nitric oxide, etc.) and non-radical species (singlet oxygen, hydrogen peroxide, lipid peroxides). These disrupt the balance of the oxidant–antioxidant defense system, including the enzymes CAT, SOD, GPX and non-enzymatic antioxidants such as GSH and total antioxidant capacity, leading to oxidative stress and damage to lipids, proteins, carbohydrates, and DNA [4].

Semen-preservation methods are broadly classified into three categories: room temperature storage (15–25 °C), chilled liquid storage (0–5 °C), and cryopreservation in liquid nitrogen (−196 °C). During the liquid preservation of ram semen at chilling temperature (0–5 °C), sperm are usually preserved in a basic extender containing Tris, citric acid, fructose, antibiotics, and distilled water at refrigerated temperatures for several days. However, the fertility of chilled or fresh ram sperm post-liquid-preservation declined rapidly with an average decrease of 10–35% per 24 h, which limits and restricts the maximum distance between the sire location and place of insemination [2]. For the chilling storage of ram sperm, diluted semen samples were first wrapped in a cotton or towel and kept at room temperature (20–25 °C) for 60 min. These samples were then cooled slowly to a chilling temperature (0–5 °C) in a refrigerator to prevent cold shock damage and preserve sperm quality parameters [5,6].

During the cryopreservation of ram semen at (−196 °C), various concentrations of glycerol and egg yolk were added to a basic Tris-based extender, which maintained sperm quality for several months or even years during cryo-storage. Sperm cryopreservation involves the two-step dilution of the semen sample with diluents and equilibration at 5 °C for 4 h. Semen samples were then packed into 0.25 mL or 0.5 mL straws sealed with polyvinyl alcohol (PVA) powder and placed horizontally at 5 cm above liquid nitrogen vapors for 20 min. Semen straws were then plunged into a liquid nitrogen container and cryopreserved at −196 °C, which suppresses sperm metabolism and biological activities without loss of functionality to overcome the constraint of time and location of use [7,8]. Naturally, ram seminal plasma comprises various enzymatic (CAT, SOD, GPx, G.R, etc.) and non-enzymatic antioxidant constituents (vitamins, zinc, selenium, carotenoids, albumin, taurine, etc.), but their shielding effect against oxidative stress damage was significantly reduced during subsequent semen dilution in the extender before the preservation process [9].

In defective spermatozoa, approximately 80–90% of ROS are produced at the mid-piece/mitochondrial site. However, at physiological levels, ROS are also generated in the plasma membrane and play an important role in sperm functioning, including capacitation, the acrosome reaction, and fertilization [10,11]. Decreased sperm cell functioning caused by OS ultimately results in reduced sperm motility indices, vitality, and structural and membrane integrity, leading to reduced fertility rate, resulting in decreased production [12,13]. Additionally, the ovine sperm plasma membrane is rich in polyunsaturated fatty acids (PUFAs), making it highly liable to freezing damage and LPO during the preservation process. Although the essential antioxidant defense systems like SOD, GPx, PRDX, GSH, and Vit E, as well as taurine, are present inside the sperm cells, artificial insemination in ovine is very limited due to the short, effective life of sperm caused by the overproduction of ROS and consequent damage during preservation [14].

Exogenous antioxidant supplementation plays an important role in maintaining higher sperm quality characteristics during the liquid storage and cryopreservation of ram semen [15]. Oxidative stress due to excessive ROS generation and nitrosative stress due to increased reactive nitrogen species production during semen preservation can adversely affect sperm quality, and exogenous antioxidant supplementation in the extender helps combat this by decreasing the destructive effects of free radicals and lipid peroxidation [16,17]. Previous studies show that several antioxidants have been added to basic semen extenders to improve sperm quality parameters in ovine semen, including lycopene [18], taurine [19], astaxanthin [20], Coenzyme Q10 [21], chlorogenic acid [22], vitamins C and E [23], punicalagin [24], puerarin [25], and alpha-lipoic acid [26]. These compounds have antioxidant properties and have been supplemented to improve sperm physiological and biochemical characteristics, further protecting against OS damage. Their supplementation ultimately led to alleviating ram semen infertility problems and improving reproductive outcomes in assisted reproductive technology in the sheep production industry [27,28].

Literature Search Strategy: There is no prior comprehensive study on various sperm-quality damages during the liquid storage and cryopreservation of semen from various ram breeds, including the role of non-enzymatic antioxidant substances as cryoprotective agents in the ram diet, in extender, or during the preservation process. Therefore, the authors broadly study and assemble various previously published (1994–2026) original research and review articles. Various search tools like Google Scholar, PubMed, Scopus, Web of Science, and Medline were used to collect the databases used in that review article. In total, 172 peer-reviewed original research articles and reviews addressing ram semen preservation were included in this review.

Inclusion criteria: (1) original research or review articles; (2) studies on ram/sheep semen during liquid storage or cryopreservation; (3) studies evaluating non-enzymatic antioxidants in diet, extender, or preservation media; (4) articles published in English.

Exclusion criteria: (1) studies on other species without ram-specific data; (2) conference abstracts, theses, and editorials; (3) articles without full text access.

Two authors independently screened titles, abstracts, and full texts. Disagreements were resolved by discussion with a third reviewer. Data on species, breed, antioxidant type, dose, extender, storage method, and sperm quality outcomes were extracted into a standardized form. Study quality was assessed using Systematic Review Centre for Laboratory animal Experimentation (SYRCLE)’s risk of bias tool for animal studies.

## 2. Effect of Liquid Storage or Cryopreservation on Sperm Morphological, Physiological and Functional Characteristics

**Figure 1:**
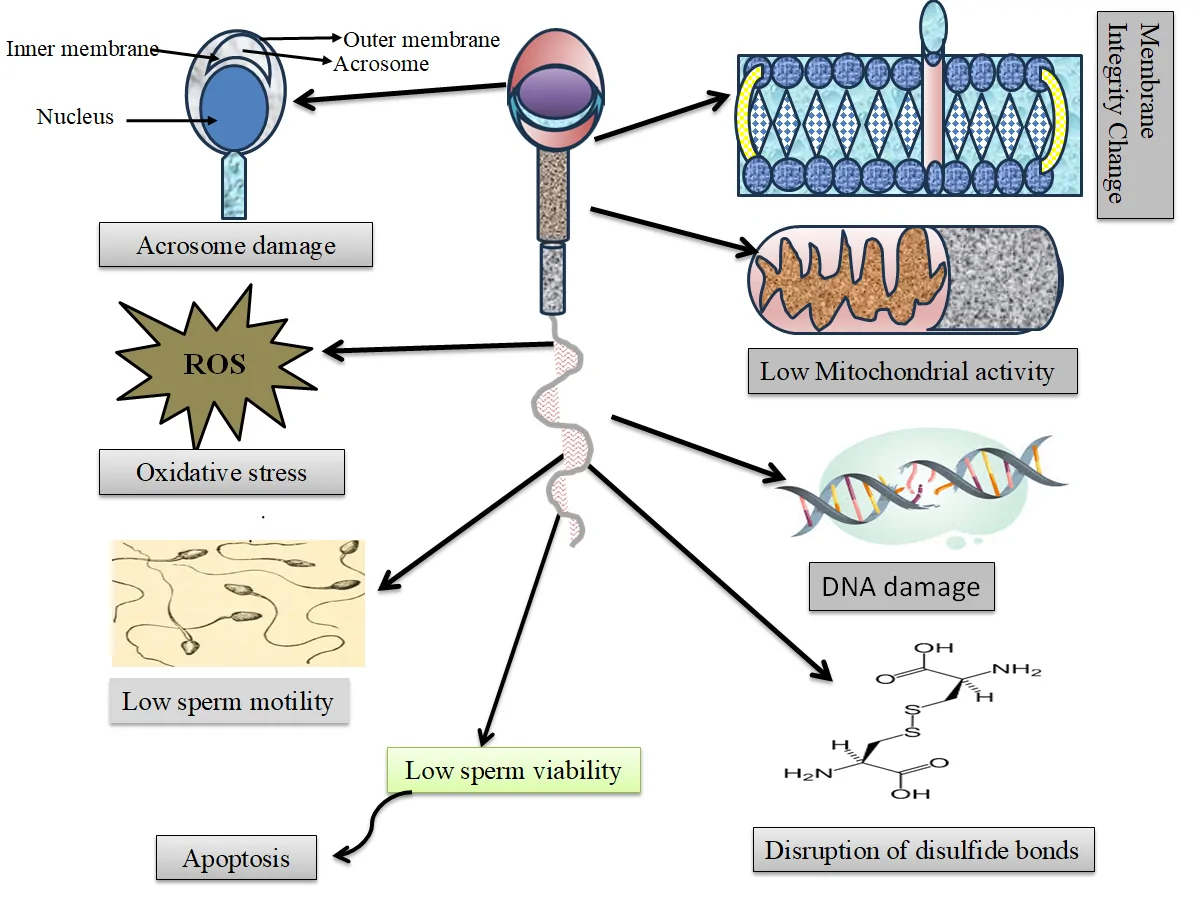
Structural and functional changes that ram sperm experience during liquid storage and cryopreservation.

Semen preservation (liquid storage or cryopreservation) is associated with a reduction in sperm physiological, biochemical, and fertility characteristics. These ultrastructural, biochemical, and functional alterations result in impaired transport and a decline in sperm survival within the female reproductive system, leading to decreased fertility [29,30]. The liquid or freezing storage of ram semen is associated with protein changes on the sperm membrane surface. Abrupt changes in temperature during preservation and cell dehydration boost specific changes in the sperm membrane’s basic components and the loss of cholesterol and fatty acids, resulting in increased metabolic activity of sperm and the start of the hyperactivation and capacitation process [31]. This reformation damages the permeability of the sperm membrane to ions, water, and cryo-protectants, leading to harm to mitochondrial activity, sperm DNA, plasma membrane, and acrosomal integrity during the preservation process, as shown in Figure 1 [32].

### 2.1. Effect of ROS on Sperm Quality Parameters

During semen quality evaluation tests, sperm motility is a main index as it is positively associated with fertility, along with DNA integrity and mitochondrial activity [33]. Certainly, efficient ram semen preservation is linked with an irreversible decrease in sperm quality parameters and metabolic activity following preservation [34]. ROS are generated when aerobic conditions are involved during live sperm cell preservation. Excessive ROS deposition due to higher production leads to OS following LPO, which provokes an irreversible loss of viability, motility, and membrane integrity, along with inhibition of respiration and fructolysis in ram sperm cells. This could be a justification for the decline in sperm quality characteristics observed during the preservation process [35,36]. Also, sperm motility is predominantly related to mitochondrial activity, as mitochondria represent the sperm energy producer alongside the major site of intracellular ROS creation, resulting in an interruption of the electron transport chain (ETC). This interruption also contributes to the decline of motility and intensifies lipid peroxidation in the sperm membrane structure [37].

**Figure 2:**
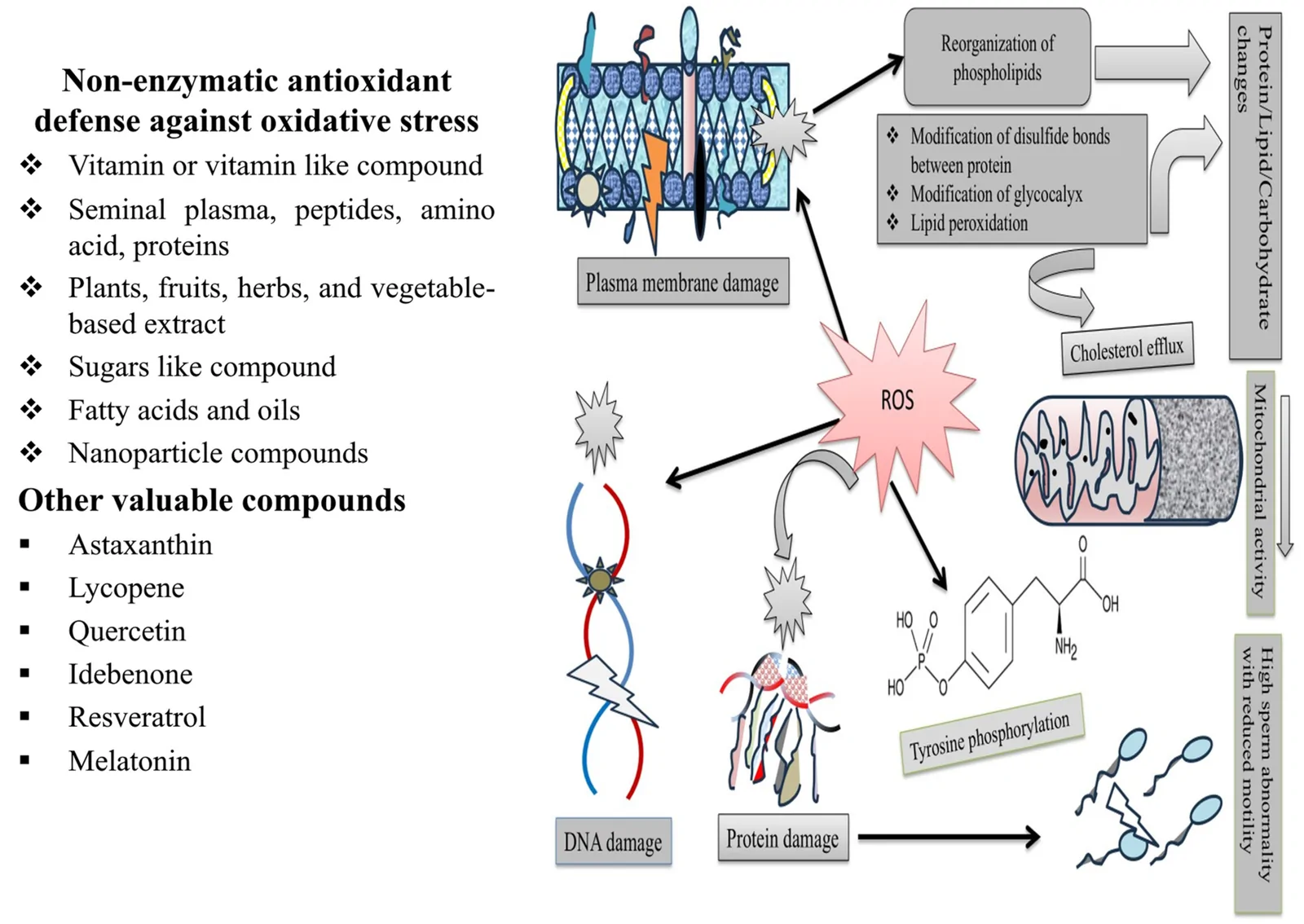
Effect of oxidative stress on ram sperm quality parameters such as plasma membrane structure, mitochondrial activity, protein and DNA structure, tyrosine phosphorylation, and motility index during liquid storage or cryopreservation with the role of non-enzymatic antioxidants.

Reorganization of sperm membrane components during storage-associated processes alters lipid–carbohydrate, lipid–protein, and protein–carbohydrate bindings necessary for appropriate membrane functionality. Excessive ROS production disrupts protein, lipid, and carbohydrate structure in the sperm membrane, breaks down disulfide bonds, and causes lipid peroxidation, along with modifications of the glycocalyx. The sperm membrane becomes fragile with loss of permeability and integrity. It also causes sperm DNA and mitochondrial structure damage, tyrosine phosphorylation, and impaired axonemal structure, which negatively affect mitochondrial activity, axonemal and DNA integrity, resulting in the loss of sperm motility as shown in Figure 2 [9].

### 2.2. Source of ROS in Semen Samples

Intrinsic or physiological source of ROS: Sperm themselves are the main source of ROS production in semen samples. The quantity of ROS generation depends on the maturation stage. In mature sperm cells, ROS creation occurs either in the plasma membrane structure—especially H_2_O_2_ and superoxide anion by nicotinamide adenine dinucleotide phosphate oxidase—or in the presence of a nicotinamide adenine dinucleotide-dependent oxidoreductase in the mitochondrial inner membrane, which confirms ROS formation through electron leakage from the electron transport chain (ETC) [38,39]. At physiological concentrations, ROS are required for sperm capacitation, hyperactivation, and the acrosome reaction. However, cooling, chilling, and cryopreservation induce excessive ROS production, shifting levels to a pathological range.

Pathological source of ROS: The leukocytes found in the seminal plasma are the second main source of ROS generation. Leukocytic production is enhanced during preservation to counter infectious agents, leading to leukocytospermia; reduced production of superoxide dismutase (SOD) results in excessive ROS production, leading to oxidative stress (OS), lipid peroxidation, DNA fragmentation, and reduced sperm quality [36].

In dead or abnormal sperm, damage to the mitochondrial membrane causes leakage of ROS and their overproduction. In the sperm plasma membrane, abrupt temperature changes during chilled storage or cryopreservation cause membrane phase transitions, Fe^2+^/Cu^2+^ release, and hydroxyl radical production. The removal of seminal plasma during sperm washing and centrifugation reduces natural antioxidant content and leads to ROS accumulation. Additionally, some cryoprotective agents, such as egg yolk and glycerol, undergo auto-oxidation during storage and generate excessive ROS. Metal ions from water, glassware, and extenders can catalyze ROS production through oxidation reactions.

Extrinsic sources: Excessive ROS production in the semen might be associated with several extrinsic factors, including aging, heat stress, toxicants or radiation exposure, nutritional deficiencies, and environmental factors. The ROS content in semen samples also depends on in vitro techniques used for sperm cell washing and preservation. Pelleting spermatozoa by a series of centrifugations and resuspension promotes extensive ROS production [39].

#### Different Methods Used for ROS Detection in Spermatozoa

Dichlorodihydrofluorescein diacetate (DCFH-DA/DCF): DCFH-DA is a cell-permeable probe. After deacetylation and oxidation, it yields fluorescent DCF, which can be measured by flow cytometry as an indicator of overall intracellular ROS levels, including H_2_O_2_.

Dihydroethidium (DHE): It detects intracellular superoxide radicals. DHE fluorescence increases after freeze-thawing due to elevated ROS production from damaged mitochondria.

MitoSOX Red: It targets mitochondria and detects superoxide radicals specifically from the sperm midpiece.

Luminol and Lucigenin. These are chemiluminescent probes used to measure extracellular ROS. Luminol detects H_2_O_2_ and HOCl from leukocytes, while lucigenin is more specific for superoxide. Signals are typically high in semen with elevated leukocyte levels due to pathological conditions.

### 2.3. Defense Against ROS in Semen Samples

Antioxidants are enzymes or compounds that can dispose of, scavenge, counter, and thwart the formation of ROS. These help retain sperm cell morphology or function by shielding the acrosomal membrane and the functional membrane against reactive oxygen species, thereby preventing premature acrosomal reaction [40]. Limited or reduced ROS generation due to antioxidant addition into basic diluent during the semen storage process also inhibits sperm cell DNA fragmentation and cryo-damage to improve their quality characteristics [41]. During spermatogenesis, sperm lose most of their intracellular cytoplasmic substances, resulting in limited antioxidant capacity and defense against ROS damage. Seminal plasma functions as the key barrier against extracellular reactive oxygen species, containing various enzymatic—catalase (CAT), superoxide dismutase (SOD), glutathione peroxidase (GPx), glutathione reductase (G.R), peroxiredoxin (PRDX)—and non-enzymatic antioxidant substances including carotenoids (vitamin A), coenzyme Q10, reduced glutathione (GSH), pyruvate, taurine, hypo-taurine, vitamin C, uric acid, and vitamin E [16,42].

Although ram sperm possess endogenous enzymatic and non-enzymatic antioxidant defenses, these systems are inherently limited and become critically compromised under semen cryopreservation conditions due to dilution, cold inactivation, and removal of seminal plasma. Simultaneously, cryopreservation induces a surge in ROS production from damaged mitochondria and dead sperm. This creates an oxidative stress gap that endogenous defenses cannot close. Therefore, supplementation of semen extenders with exogenous antioxidants is essential to restore antioxidant capacity, prevent lipid peroxidation and DNA damage, and maintain post-thaw sperm quality and fertility. The antioxidant defense system of the whole organism at systemic level is affected by the dietary intake of various antioxidant substances including minerals and vitamins. The use of antioxidants to defuse the overproduction of ROS either directly into the semen extenders or in addition to the diet has been well studied and described in previous studies [28,43]. In general, dietary antioxidant intake requires long-term and persistent treatment approaches to get better fertility outcomes. The effect of each antioxidant depends on the dose and species of animal used. Likewise, to preserve the membrane integrity of spermatozoa during freeze–thaw processes, various associations and mechanisms have been developed [44,45]. However, a deep understanding of how antioxidants provide protection and energy for sperm cell functioning is still contradictory.

## 3. Effect of Various Non-Enzymatic Antioxidant Supplementations

### 3.1. Supplementation of Vitamins or Vitamin-like Compounds

Vitamins supplementation in semen extender is responsible for playing an essential role in preserving sperm quality parameters along with fertility rate post-thaw [46].

Vitamin E: Ram sperm quality can be preserved by adding vitamin E to basic semen extender. It is a strong scavenger of peroxyl radicals and possibly the most vital inhibitor of the lipid peroxidation chain reaction. The addition of vitamin E (1 mg/mL) in the Tris–glucose–egg yolk-based extender improved sperm motility and kinematic parameters but did not affect viability and membrane integrity during the chilling storage of Kail ram sperm for 72 h [23]. Vitamin E-loaded cyclodextrins supplementing the freezing medium had a significant positive effect on ram epididymal sperm motility indexes and membrane integrity, along with reduced oxidative stress and lipid peroxidation damage as shown in Table 1 [47].

Trolox is an analog of vitamin E, highly soluble in water, that acts as a scavenger of lipid peroxyl radicals through LPO chain-breaking action. Its defensive effect against lipid peroxidation and H_2_O_2_ generation has been described for ram semen [14]. Further, supplementing Vitamin E (Trolox) at 60 and 120 μM to a semen-freezing extender during cryopreservation preserved the sperm ultrastructural integrity, mitochondrial potential, and motility parameters, with enhanced sperm survival post-thaw [48]. The use of Trolox during liquid storage of ram semen at 5 °C and 15 °C negatively affects sperm quality, suggesting that the storage temperature and extender composition affect its action. It could be concluded that efficacy of Vitamin E and Trolox supplementation is temperature-dependent because their solubility, membrane incorporation, and reaction rates vary with temperature as shown in Table 1 [49].

Vitamin C, known as ascorbic acid, is soluble in water and associated with the maintenance of the sperm cells’ membrane integrity by inhibiting OS damage to sperm DNA structure. Supplementation of ascorbic acid (Vitamin C) 3 mg/100 mL of Tris–egg yolk (TEY)-based extender shows significant improvement in sperm quality parameters and viability, with reduced structural abnormalities in post-thaw Sapudi ram semen [50]. Ascorbic acid or vitamin C supplementation (0.5 mM) into gum Arabic and Tris–egg yolk maintained higher sperm viability, kinematic and biokinetic parameters, membrane/acrosomal integrity, with lower morphological defects in post-cryopreserved Noemi ram semen [51]. Alongside this, it has been observed that chilled ram semen preserved with vitamin C (4.5 mg/mL) for 72 h negatively affects sperm quality factors except for acrosome integrity, signifying that vitamin C supplemented at a lower dose is more proficient than higher concentrations. Hence, Vitamin C exhibits a biphasic dose-response in ram semen preservation. At low physiological concentrations, it acts as an efficient aqueous-phase antioxidant and recycles Vitamin E. However, at higher concentrations, Vitamin C becomes pro-oxidant by reducing free radicals’ transition, thereby promoting hydroxyl ion generation. Additionally, high doses alter extender pH and osmolarity, causing direct sperm toxicity as shown in Table 1 [52].

Vitamin B12: Supplementation of Vitamin B12 (2 mg/mL) into the freezing extender maintained higher sperm quality characteristics and morphology in cryopreserved Dallagh ram semen [53]. Another study on Arabi rams reported improvement in sperm quality, morphology, and antioxidant capacity, along with enzyme (CAT, GPx) activity following 1–2 mg/mL Vit B12 and 0.5 mg/mL Vit B1 addition into cryopreservation medium as shown in Table 1 [54].

Vitamin D: Vitamin D acts as both a hormone and antioxidant in semen. It works through genomic (regulates genes for antioxidant enzymes, calcium channels, and anti-apoptotic proteins) and non-genomic pathways (triggers calcium influx and phosphorylation cascades) that protect sperm during storage. Vitamin D (50 ng/mL) addition into the freezing medium improves sperm motility indices, membrane integrity, and mitochondrial potential, with reduced DNA fragmentation in post-thaw Merino ram semen as shown in Table 1 [55].

Coenzyme Q_10_: It is a vitamin-like substance synthesized from tyrosine and acts as an important constituent of the mitochondrial membrane structure, an energy-stimulating agent by supporting the mitochondrial ETC, present in the midpiece of the spermatozoa tail. Supplementing COQ10 (50 μmol/L) into a basic extender improves sperm biokinetic indexes, sperm morphology, antioxidant capacity, sperm MMP with ATP content, antioxidant enzyme (SOD, CAT) activity, and reduces LPO and ROS damage during room-temperature preservation of Hu sheep semen for five days [21]. Another study reported improvement in sperm viability, motility, MMP, and morphological properties with reduced sperm abnormality, lipid peroxidation, and apoptotic-like changes following 2 μM COQ10 addition into plant (soya lecithin)- and animal (egg yolk)-based semen extender during cryopreservation of ram semen as shown in Table 1 [56].

Myo-inositol: A previous study demonstrated that ultrasonic vibration (USV) of basic semen-freezing extender with MYO supplementation (7 mM) maintained higher sperm viability, kinematic factors, antioxidant capacity, mitochondrial and functional membrane integrity, with reduced lipid peroxidation up to 72 h in Mehraban ram semen post-thaw as shown in Table 1 [57].

### 3.2. Seminal Plasma, Peptides, Amino Acids, and Protein Supplementation

Amino acids are non-enzymatic antioxidant substances present in ram seminal plasma in adequate amounts [46]. Supplementing semen extenders with amino acids (e.g., cysteamine, cysteine, taurine, dithioerythritol, and methionine) had a constructive effect on spermatozoa quality indices. The supplementation of amino acids, L-glutamine (20 mM) and L-Proline (25 mM), into a Tris-based freezing extender maintained higher sperm motility, vitality, and acrosomal integrity, along with a significant decline in lipid peroxidation, in pre- and post-thaw Bannur ram semen [58]. The supplementation of glutamine (5 mM) into a Tris-based freezing extender maintained higher sperm motility, membranal/acrosomal integrity, CAT activity, along with no significant effect on lipid peroxidation and SOD activity of post-thaw Akkaraman ram semen [59]. The combined supplementation of L-glutamine (40–80 mM) and glycerol (5–7%) into Tris–egg-yolk-based freezing extender maintained higher post-thaw sperm quality parameters like motility, viability, and functional membrane integrity [60]. Dietary L-citrulline supplementation (12 g/day) for 90 days improved sperm density, sperm CASA parameters, total antioxidant content (T-AOC), SOD, CAT, GPx levels, hormonal profile, antifreeze protein, and testicular spermatogenic cell development, as well as the metabolomic and transcriptomic profiles of Turpan black ram semen as shown in Table 2 [61,62,63].

Cysteine: A low-molecular-weight amino acid containing a group of thiols, which play an important role in glutathione biosynthesis and help alleviate sperm damage due to inhibition of toxic oxygen metabolites initiated by lipid peroxidation. The supplementation of cysteine (1 mg/mL) into the freezing extender maintained higher sperm motility, vitality, acrosomal integrity, and sperm penetration in artificial vaginal mucus. Oppositely, cysteine supplementation significantly reduced ROS production in pre- and post-thaw Texel ram semen [64]. Cysteine supplementation (0.5 or 1 mM) into a gum-Arabic and a Tris–egg yolk-based extender maintained higher sperm viability, kinematic and biokinetic parameters, plasma membrane and acrosomal integrity, with lower morphological defects in post-cryopreserved Noemi ram semen [51]. Another study revealed that cysteine (1 and 2 mM) supplementation enhanced sperm motility indices, membrane integrity, and DNA integrity of Kivircik ram sperm post-thaw and post-incubation period as shown in Table 2 [65].

Cysteamine: Cysteamine supplementation (5, 10 mM) into a freezing extender improves post-thaw sperm quality parameters in Akkaraman ram semen [66]. Contrary to that, another study reported a detrimental effect of the addition of 5 mM cysteamine into freezing medium on the cryo-survival of post-thaw ram semen [67]. The contradictory result might be due to the reason that cysteamine efficacy depends on the breed-specific redox environment. Variations in endogenous GSH levels, seminal plasma protein content, sperm membrane PUFA composition, and mitochondrial susceptibility to cryo-injury dictate whether cysteamine functions as an antioxidant or pro-oxidant. Cysteamine (6 mM) addition into a soya-lecithin-based extender improves post-thaw sperm motility and membrane functionality, with significantly reduced MDA content in Mehraban ram semen [68]. Similarly, when supplementing 1 or 2 mM of cysteamine as a preservative to a basic Tris-based extender during chilling storage at 5 °C for 72 h, an increase in Merino ram sperm motility, viability, mitochondrial function, and glutathione level has been documented along with a reduction in oxidative stress [69]. Supplementing 1 μg/mL of cysteamine loaded on bacterially synthesized selenium nanoparticles (Se-NPs) as a preservative to a basic freezing extender during cryopreservation caused an increase in Sanjabi ram sperm motility, membrane integrity, viability, DNA integrity, and SOD level along with a reduction in apoptosis rate and MDA content post-thaw as shown in Table 2 [70].

Methionine: It acts as a precursor amino acid for glutathione synthesis, which helps in the protection of sperm cells against oxidative stress and cryo-damage during the semen-preservation process. Methionine (1 mM) treatment improves sperm motility indexes, although no significant effect was observed on LPO, GPx, and GSH activities during preservation [71]. It has been revealed that methionine (2 and 4 mM) supplementation enhanced the sperm motility indices, viability, and mitochondrial activity of Merino ram sperm during chilling storage at 5 °C for 4 days [72]. Another study revealed that methionine (2.5 and 5 mM) supplementation enhanced the sperm motility, membrane integrity, and DNA integrity of Kivircik ram sperm during cryopreservation and incubation time points [65]. Dietary methionine supplementation can alter and modify the sheep seminal plasma (SP) protein, proteomic and miRNA composition, which can play a potential role in reproduction, fertility, and early embryonic development as shown in Table 2 [73].

Taurine: The beneficial effects of taurine as an antioxidant in semen-preservation systems have been attributed to its ability to stabilize sperm biological membranes, scavenge ROS, and to decrease the lipid peroxidative damages post-thaw. Taurine supplementation (20 mM) into a Tris-based extender improves sperm kinematic and biokinetic parameters, membrane integrity, MMP, and antioxidant status (TAOC, SOD, CAT), with a significant reduction in ROS and MDA content during the 15 °C preservation of Hu ram semen for five days [19]. Taurine (50 mM) delivered a substantial improvement in sperm motility, survival, and membrane integrity during the 6 h chilling storage of Chios ram semen at 5 °C [74]. Taurine (40 mM) addition into a freezing medium showed improvement in post-thaw sperm quality and acrosomal and membrane integrity, along with a reduction in the MDA content of cryopreserved crossbred ram semen [15]. Hypo taurine (5 mM) addition into freezing medium showed improvement in post-thaw sperm CASA parameters, mitochondrial activity, acrosomal and membrane integrity, along with a reduction in the DNA damage of cryopreserved Merino ram semen [75]. Supplementing a lower dose of taurine (25 mM) caused an improvement in sperm motility indices, acrosomal and functional membrane integrity, along with a reduction in sperm abnormalities after 72 h conservation at 4 °C as shown in Table 2 [52].

Carnitine: L-Carnitine (5 mM) addition into a plant-based freezing medium shows an improvement in post-thaw sperm CASA parameters, mitochondrial activity, viability, pregnancy rate, acrosomal and membrane integrity, along with a reduction in apoptosis rate, LPO, ROS content, and DNA fragmentation of cryopreserved Zandi ram semen [76]. L-Carnitine supplementation (5 mM) into a skimmed-milk–egg yolk-based extender improves sperm CASA parameters, plasma/acrosome/mitochondrial membrane integrity, and fertilization potential during the (5–15 °C) preservation of chilled ram semen for 96 h [77]. L-Carnitine (5 or 10 mM) addition into two different extenders, Tris–egg yolk based or commercial freezing medium, shows an improvement in post-thaw sperm CASA parameters, capacitation status, and membrane integrity along with a reduction in LPO and oxidative stress of cryopreserved ram semen as shown in Table 2 [78].

Bovine serum albumin: Optimal bovine serum albumin (10%) addition into a Tris-based extender improves sperm CASA parameters, plasma/acrosome membrane integrity, and viability, along with a reduction in MDA content during 4 °C preservation of Zandi ram epididymal sperm for 120 h [79]. Another study reported that (5 mg/mL) BSA addition into a Tris-based extender improves sperm CASA parameters, plasma/acrosome membrane integrity, and antioxidant (GSH) capacity, along with a reduction in MDA content during 5 °C preservation of chilled Kivircik ram semen for 72 h [80]. Bovine serum albumin (10 or 15%) addition into a Tris-based extender improves sperm CASA parameters, membrane integrity, and viability, along with a reduction in morphological defects during cryopreservation of ram semen [81]. Bovine serum albumin column helps in the sex sorting of ram spermatozoa through sperm kinematic properties, DNA quantification, enzyme activity, and the toll-like receptor (TLR)7/8 ligand R848 application protocol as shown in Table 2 [82].

Kinetin: Kinetin (50 and 100 µM) addition into basic extender improves sperm CASA parameters, plasma membrane integrity, viability, and antioxidant activity along with reduction in MDA content during chilling preservation of Qezel ram semen for 72 h as shown in Table 2 [83].

**Table 2:** Effects of different amino acids, peptides, or protein-like compound supplementation on ram sperm quality parameters during liquid storage or cryopreservation.

Antioxidants	Optimal Conc.	Temp	Time	Ram Breed	Results	Ref.
L-Glutamine L-Proline	20 mM 25 mM	−196 °C	Pre- & post-thaw	Bannur	Improved sperm motility, viability, PMI, and ACI with reduced lipid peroxidation.	[58]
Glutamine	5 mM	−196 °C	Post-thaw	Akkaraman	Improved sperm motility, PMI, and CAT activity with no significant effect on MDA and SOD activity.	[59]
L-Glutamine Glycerol	80 mM 7%	−196 °C	Post-thaw	N/A	Improved sperm motility, viability, and functional membrane integrity.	[60]
L-Citrulline	12 g/d orally		After 90 days	Turpan black	Improved semen quality, CASA parameters, PMI, ACI, antifreeze protein, CAT, TAC, NO, GPx, testicular histology, ruminal flora, and HSP90 expression.	[61]
L-Citrulline	12 g/d orally		After 72 days	Turpan black	Improved protein digestion, testicular gene regulation, seminal amino acid content, and sperm quality.	[62]
L-Citrulline	12 g/d orally		After 70 days	Turpan black	Improved sperm motility, viability, PMI, MMP, TAC, SOD, CAT, NO, GPx, FSH, LH, and GnRH levels. Reduced MDA, H_2_O_2_, OH, E_2_ and O_2_^−^ level.	[63]
Cysteine	1 mg/mL	−196 °C	Pre- & post-thaw	Texel	Improved sperm motility, viability, ACI, and penetration in artificial cervical mucus with reduced ROS levels.	[64]
Cysteine	1, 2 mM	−196 °C	Post-6 h incubation & post-thaw	Kivircik	Enhanced sperm motility, plasma membrane/DNA integrity.	[65]
Cysteine	0.5, 1 mM	−196 °C	Post-thaw	Noemi	Improved sperm motility/CASA parameters, viability, PMI, and ACI with reduced sperm abnormality.	[51]
Cysteamine	5 mM	−196 °C	Post-thaw	Akkaraman	Improved sperm motility with no effect on PMI, ACI, CAT, MDA, GSH, and GPx activity.	[66]
Cysteamine	5 mM	−196 °C	Post-thaw	N/A	Negative effects on cryo-survival of post-thaw ram sperm.	[67]
Cysteamine	6 mM	−196 °C	Post-thaw	Mehraban	Improved sperm motility, viability, and PMI with reduced MDA content. No effect on sperm P.M, capacitation, GPx activity, and abnormality.	[68]
Cysteamine	1, 2 mM	5 °C	72 h	Merino	Improved sperm motility, viability, mitochondrial function, and total glutathione level with reduced oxidative stress.	[69]
Cysteamine loaded Se-NPs	1 µg/mL	−196 °C	Post-thaw	Sanjabi	Improved sperm motility, viability, PMI, DNA integrity, and SOD activity with reduced sperm abnormality, apoptosis rate, and MDA content.	[70]
Methionine	1 mM	5 °C	72 h	Merino	Improved sperm motility indices with no significant effect on LPO, GPx, and GSH activities	[71]
Methionine	2, 4 mM	5 °C	96 h	Merino	Improved sperm motility, viability, and mitochondrial activity	[72]
Methionine	2.5, 5 mM	−196 °C	Post-6 h incubation & post-thaw	Kivircik	Enhanced sperm motility and plasma membrane/DNA integrity.	[65]
Taurine	20 mM	15 °C	5 days	Hu	Improved sperm kinematic and biokinetic parameters, PMI, MMP, TAC, SOD, and CAT activity with a significant decline in ROS and MDA content.	[19]
Taurine	50 mM	5 °C	30 h	Chios	Improvement in sperm motility, survival, and membrane integrity.	[74]
Taurine	40 mM	−196 °C	Post-thaw	crossbred	Improved sperm quality, PMI, and ACI with reduced MDA content.	[15]
Hypo-taurine	5 mM	−196 °C	Post-thaw	Merino	Improved sperm viability, CASA parameters, PMI, ACI, and mitochondrial activity with reduced DNA damage.	[75]
Taurine	25 mM	4 °C	72 h	N/A	Improved sperm motility, viability, PMI, and ACI with reduced morphological abnormalities.	[52]
L-Carnitine	5 mM	−196 °C	Post-thaw	Zandi	Improved sperm CASA parameters, MMP, viability, pregnancy rate, PMI, and ACI with reduction in apoptosis rate, LPO, ROS content, and DNA damage.	[76]
L-Carnitine	5 mM	5–15 °C	96 h	N/A	Improved sperm CASA parameters, PMI, ACI, mitochondrial membrane integrity, and fertilization potential.	[77]
L-Carnitine	5, 10 mM	−196 °C	Post-thaw	N/A	Improved sperm CASA parameters, capacitation status, and membrane integrity with reduced LPO and oxidative stress.	[78]
Bovine serum albumin	10%	4 °C	120 h	Zandi	Improved sperm CASA parameters, PMI, ACI, and viability with reduced MDA content.	[79]
Bovine serum albumin	5 mg/mL	5 °C	72 h	Kivircik	Improved sperm CASA parameters, PMI, ACI, GSH, and TAC with reduced MDA content.	[80]
Bovine serum albumin	10, 15%	−196 °C	Post-thaw	N/A	Improved sperm CASA parameters, PMI, and viability with reduced morphological defects.	[81]
Kinetin	50, 100 µM	4 °C	72 h	Qezel	Improved sperm CASA parameters, PMI, viability, and TAC, not affecting SOD and NO levels with reduced MDA content.	[83]
Antifreeze protein type 1	0.1, 0.5 μg/mL	−196 °C	Post-thaw	N/A	Improved sperm CASA parameters and PMI, having no effect on ACI, MMP, MDA, and DNA integrity with reduced morphological defects.	[84]
Bioactive peptide	60 μg/mL	−196 °C	Post-thaw	Han	Improved sperm motility, PMI/ACI, vitality, CAT, SOD, GPx activity, in vitro fertility, and cleavage rate with reduced MDA and ROS content.	[85]

Note: CASA: Computer-assisted sperm analysis; CAT: Catalase; PMI: Plasma membrane integrity; SOD: Superoxide dismutase; LPO: Lipid peroxidation; ACI: Acrosomal integrity; GPx: Glutathione peroxidase; H_2_O_2_: Hydrogen peroxide; TAC: Total antioxidant contents; HSP90: Heat shock protein-90; NO: Nitric oxide; GSH: Reduced glutathione; FSH: the; GSH: Reduced glutathione; LH: Luteinizing hormone; GnRH: Gonadotropin-releasing hormone; E2: Estradiol; OH: Hydroxyl level; ATP: Adenosine triphosphate; ROS: Reactive oxygen species; MMP: Mitochondrial membrane potential; MDA: Malondialdehyde.

Antifreeze protein type 1: Antifreeze protein (0.1 to 0.5 μg/mL) addition into the Tris-based freezing extender improves sperm cryo-resistance, CASA parameters, and membrane integrity, along with a reduction in morphological defects during the cryopreservation of ram semen [84]. Bioactive peptide (BAPT 60 μg/mL) addition into the Tris-based freezing medium shows improvement in post-thaw sperm motility, plasma/acrosome membrane integrity, vitality, CAT, SOD, GSH-PX activity, in vitro fertility, cleavage rate, along with reduction in MDA, ROS content of cryopreserved Han ram semen [85]. Another study proved that seminal plasma (SP) proteins that bound to the sperm membrane were preserved among ram breeds during the cryopreservation of semen; this SP protein, when supplemented to frozen/thawed semen extender along with an energy source, repaired Texel ram sperm damage and improved sperm quality [86]. Binder of sperm protein types (BSP1 and BSP5) isolated by gelatin affinity and purified by chromatography improve ram sperm post-thaw motility, membrane integrity, and penetration through mucus along with reduction in tyrosine phosphorylation as shown in Table 2 [87].

### 3.3. Supplementation with Plant, Fruit, Herb, and Vegetable-Based Extracts and Polyphenols

There has been an increasing interest in natural antioxidants found in plants, fruits, vegetables, oilseeds, and herbs to maintain higher semen quality parameters during preservation. Their phytochemical structure and recent use in the cosmetic, pharmaceutical, and food industries with promising effects make them an innovative research direction. Because of their powerful antioxidant ability and many biochemical compounds, various studies have examined the beneficial effect of plant extract supplementation to stop or limit storage-related damage to ram spermatozoa.

Rosemary is an herb with a complex molecular formula containing various compounds including rosmarinic acid, carnosic acid, camphor, borneol, and bornyl acetate. Their molecular formula and composition may vary depending on various factors like plant origin, extraction methods, and specific cultivars. The supplementation of rosemary extract (4 or 6%) to a soya lecithin-based semen extender shows a significant improvement in sperm motion kinetic parameters, viability, and plasma membrane integrity, having no effect on acrosome and capacitation with reduced MDA damage during the cryopreservation of Chal ram semen [88]. The supplementation of rosemary honey in place of fructose to a Tris–egg yolk (TEY)-based extender shows significant improvement in sperm quality parameters like motility, viability, membrane integrity, and embryo development rate with reduced structural abnormality in post-thaw Rasa Aragenosa ram semen [89]. The supplementation of rosemary essential oil (12.5, 25 μg/mL) to a Tris–egg yolk (TEY)-based extender shows significant improvement in sperm CASA parameters, viability, membrane integrity, and MMP with reduced oxidative stress in post-thaw ram semen [90]. The supplementation of the rosemary-derived bioactive compounds gallic acid (2 mM) and carnosic acid (0.05 mM) to a Tris-based semen extender shows significant improvement in sperm CASA parameters, plasma membrane/acrosomal integrity, and mitochondrial potential during cryopreservation of Merino ram semen as shown in Table 3 [91].

The supplementation of Moringa leaf methanolic extract (0.64 mg/mL) into a Tris–citrate-based freezing medium enhances sperm motility/viability/plasma membrane/acrosome integrity, SOD, and GPx activity, along with a significant decline in total cholesterol, low-density lipoprotein, MDA, nitric oxide, and copper content of post-thaw Awassi ram semen [92]. The supplementation of a basic ram diet with Moringa oleifera leaf extract (40 mg/kg) improved semen volume, sperm concentration, sperm motility/viability/membrane integrity, TAC, SOD, CAT, GPx, glutathione reductase, ALP, ACP, and ascorbic acid, along with a significant decline in DNA damage and MDA content during the liquid storage and cryopreservation of Barki ram semen. Moringa oleifera leaves are rich in nutrients such as protein, zinc, selenium, vitamins, and beta-carotene. Dietary supplementation with these compounds provides antioxidant protection, improves blood circulation, and increases testosterone levels, which may result in increased ram semen volume, sperm concentration, and motility indexes [93]. The supplementation of Moringa oleifera seed extract (5 &10 mg/mL) into a basic freezing medium enhances sperm motility/viability/membrane/acrosome integrity, mitochondrial/antimicrobial/antioxidant activity, along with higher in vitro fertility rate of hair ram semen [94,95]. The supplementation of Quinoa seed extract (750–1000 µg/mL) into a typical semen-freezing medium enhances sperm motility/viability/acrosome integrity, SOD1, TAC, CAT, GABPB1, and GPX1 gene expression, along with a significant decline in apoptotic changes, H_2_O_2_, MDA content and CASP3 gene expression of post-thaw Rahmani ram semen [96]. The supplementation of cinnamon extract (100 µL/mL) into a typical diluted semen sample enhances sperm CASA motility indexes/viability/acrosome integrity, and morphology during 4 °C preservation of Arabi ram semen up to 72 h [97]. The supplementation of acetone extract from Opuntia ficus indica cladodes (1%) into a Tris–egg-yolk- or skim-milk-based freezing medium enhances sperm motility/viability/plasma membrane/acrosome integrity along with a significant decline in lipid peroxidation and DNA damage during 5 °C preservation of ram semen up to 72 h [98]. The supplementation of Entada abyssinica bark extract (375 µg/mL) into a Tris–citrate-acid–soya -lecithin-based freezing medium enhances sperm motility/viability/plasma membrane/acrosome integrity and TAC, while no significant effect on ALT, AST, LDH activity, along with a significant decline in sperm necrotic and apoptotic changes in post-thaw Ossimi ram semen as shown in Table 3 [99].

The supplementation of lavender ethanolic extract (150 µg/dL) into a semen-freezing medium enhances sperm motility/viability/acrosome/membrane integrity, MMP, SOD level, fertility parameters, while not affecting GPx activity, along with a significant decline in apoptotic changes, H_2_O_2_, MDA content, and DNA damage of post-thaw shall ram epidydimal spermatozoa [100]. The supplementation of ginger and echinacea extract (10 and 20 mg/L into a semen-freezing medium enhances sperm motility/viability/acrosome/membrane integrity, mitochondrial activity, and fertility parameters, along with a significant decline in lipid peroxidation and DNA damage of post-thaw ram epidydimal spermatozoa [101]. The supplementation of hydroethanolic extracts of Terminalia chebula and Thymbra spicata (each at 3000 µg/mL) into a typical semen-preservation medium enhances sperm viability and DNA integrity, along with a significant decline in lipid peroxidation, MDA, and H_2_O_2_ content under normal and oxidative stress-induced fresh ram semen [102]. Supplementing curcumin (20 µmol/L) into typical semen-preservation medium enhances sperm viability/motility/membrane/acrosome integrity, T-AOC, CAT, SOD activity, metabolic profile, along with a significant decline in lipid peroxidation, MDA, and ROS content during 4 °C preservation of Hu ram semen as shown in Table 3 [103].

The supplementation of the best treatment of Clove bud extract (35 or 75 µg/mL) into different semen-freezing media enhances sperm viability/motility/membrane integrity and antioxidant activity of post-thaw ram semen [104]. The supplementation of fennel extract (10 mg/L) into a soya-lecithin-based extender enhances sperm viability/motility/membrane integrity and mitochondrial activity along with a significant decline in lipid peroxidation, apoptotic changes, and abnormality of post-thaw Ghezel ram semen [105]. The supplementation of a basic ram diet with Tinospora cordifolia (1 g/kg B.W) for 6 months has no considerable effect on the physiological, biochemical, seminal, and testosterone conc of Muzaffarnagari ram semen; however, SOD, CAT, and cholesterol concentration significantly increased [106]. The supplementation of oregonin (100 µM) into a Tris–glucose–egg-yolk-based freezing medium enhances sperm motility parameters evaluated by CASA/morphological parameters/mitochondrial activity and fertilization potential during 5 °C preservation of locally bred ram semen up to 48 h [107]. The supplementation of cactus seed oil (1 or 2%) into a Tris–egg-yolk- and skim-milk-based extender enhances sperm motility/viability/morphological parameters along with a significant decline in lipid peroxidation and DNA fragmentation during 5 °C preservation of Boujaad ram semen up to 72 h as shown in Table 3 [108].

The supplementation of punicalagin (pomegranate extract; 30 µM) and chlorogenic acid 0.8 mg/mL into a semen-preservation medium enhances sperm CASA indexes/membrane/acrosome integrity, T-AOC, CAT, SOD, and MMP activity, along with a significant decline in MDA and ROS content during 4 °C preservation of Hu ram semen for 5 days [22,24]. Punicalagin (pomegranate extract; 15 µM) addition into Tris-based semen-freezing medium enhances sperm CASA indexes/membrane/acrosome integrity, T-AOC, CAT, SOD, ATP, total glutathione, MMP, and Nrf2/PGC-1α pathway activity, along with a significant decline in MDA, ROS content, and sperm apoptosis during the cryopreservation of Hu ram semen [109]. The supplementation of silymarin (100 µg/mL) and caproic acid (0.312%) into a Tris–glucose-based semen-preservation medium enhances sperm viability/motility indexes/membrane/acrosome integrity, along with a significant decline in MDA content during 5 °C preservation of ram semen for 3 days [110]. The supplementation of ellagic acid (1–2 mM) and ebselen (10–40 µM) into a Tris–fructose-based semen extender enhances sperm viability/motility indexes, mitochondrial membrane potential, DNA integrity, antioxidant potential, and glutathione content along with a significant decline in lipid peroxidation during 5 °C preservation of Merino ram semen for 3 days [111]. The supplementation of quinic acid (100 µg/mL) into a Tris-based semen cryopreservation medium enhances sperm CASA indexes/viability, MMP, DNA integrity, total antioxidant status along with a significant decline in MDA, ROS content and redox parameters of post-thaw Ramlic ram semen [112]. The supplementation of acetonic and hexanoic extract of Spirulina platensis (1.25 µg/mL) and acetonic and methanolic extracts of Salvia verbenaca (3.75 μg/mL, 6.25 μg/mL) into a skim-milk-based semen-preservation medium show improvement in sperm motility indexes/viability/membrane integrity and fertility with significant decline in lipid peroxidation and sperm abnormality during 4 °C preservation of ram semen for 24 h as shown in Table 3 [113].

### 3.4. Supplementation of Ram Semen Extender with Sugars

Carbohydrates, with their various forms like monosaccharides, disaccharides, and trisaccharides, are widely used in ram semen preservation. Sugars serve as an energy source for sperm during storage and help to maintain the osmotic stability of basic semen extenders. The supplementation of sucrose, trehalose, or raffinose sugars (50–100 mM) to a Tris-based semen-freezing extender shows significant improvement in sperm motility parameters, viability, acrosome/plasma membrane integrity, and fertility rate following post-thaw ram semen, along with reduction in acrosomal and other sperm abnormalities during cryopreservation [114]. The supplementation of sucrose into a Tris–egg yolk-based extender, along with slow freezing or refrigeration of the semen sample just before vitrification, preserves the post-thaw sperm motility, morphology, membrane integrity, DNA integrity, and cryo-survival of Merino ram semen [115]. The supplementation of trehalose (100 mM), sucrose (60 mM), and raffinose (10 mM) sugars along with Vit E (2 mM) addition into a Tris–egg-yolk-based semen-freezing extender shows significant improvement in sperm quality parameters like viability/total/progressive motility during cryopreservation of the Iranian Afshari ram semen [116]. Another study in Australian Merino ram revealed that the addition of glycerol and disaccharide sugars (sucrose and trehalose) into hypertonic diluent either before or after cooling from 30°C to 5 °C is beneficial for sperm motility, plasma membrane/acrosome region integrity during cryopreservation [117]. Trehalose (50 mM) supplementation into a basic semen extender improves sperm quality parameters in terms of motility, viability, and membrane integrity, along with a significant decline in sperm abnormalities during the 5 °C preservation of Chios ram semen up to 30 h [74]. The supplementation of sugars like trehalose, sucrose, or glucose at a range of (30–210 mM) into a Tris–egg-yolk–glycerol-based semen extender improves sperm quality parameters in terms of motility, viability, acrosome integrity, and fertility following cervical or intrauterine artificial insemination with post-thaw ram semen as shown in Table 4 [118].

The supplementation of trehalose (100 mOsm) into a Tris–fructose-based freezing medium enhances sperm motility, plasma membrane/acrosome integrity, and in vivo fertility using post-thaw Pampinta ram semen [119]. The addition of trehalose (100 mOsm) in combination with dimethylacetamide (3 or 6%) reduced the sperm motility indexes; however, they have a positive effect on plasma membrane and acrosomal integrity rate during cryopreservation of Ines Santa ram semen. Lower sperm motility might be due to the reason that high concentrations of trehalose cause excessive dehydration and viscosity, impairing flagellar beat, while DMA exhibits direct mitochondrial toxicity and alters membrane fluidity in ram sperm. Also, a combination of a penetrating and non-penetrating cryoprotectant could create conflicting osmotic forces during freeze–thaw [120]. The combined use of glycerol (3%) and trehalose (60 mM) in a Tris-based freezing extender shows a synergistic effect with improvement in sperm motility, viability, acrosome integrity, and mitochondrial activity during the cryopreservation of Merino ram semen [121]. Trehalose supplementation modifies the proteomic profile of ram spermatozoa during cryopreservation to show antioxidant activity, glycolysis involvement, and cryotolerance to various stresses [122]. Trehalose (100 mM) and low-density lipoprotein supplementation into a Tris–glycerol–egg yolk-based freezing extender preserve post-thaw sperm quality parameters of Crioulo ram semen similar to conventional cryoprotectant during cryopreservation [123]. The combination of trehalose (100 mM) and glycerin (5%) supplementation into a soya-lecithin-based extender shows a synergistic effect with a significant improvement in sperm motility CASA parameters, membrane integrity, capacitation status, and mitochondrial activity, with a significant decline in apoptotic changes, abnormality, and MDA content of post-thaw Zandi ram semen as shown in Table 4 [124].

The combined supplementation of trehalose and EDTA into a freezing extender shows beneficial synergy on post-thaw sperm motility, acrosome integrity, and cryo-survival of ram spermatozoa, probably by removing calcium from the medium and avoiding cation competition with trehalose for membrane-binding sites during cryopreservation [125]. The supplementation of trehalose (100 mM) into a basic freezing extender together with a slow cooling rate during the cryopreservation of Akkaraman ram spermatozoa shows significant improvement of post-thaw sperm motility, viability, catalase activity, and membrane integrity along with no effect on MDA content, GSH, and GPx activity [66,126]. The combined supplementation of protease inhibitor antipain (10 µM) and trehalose (30 or 60 mM) into a freezing medium exhibits a synergistic effect and significant improvement of post-thaw total and progressive motility of sperm, morphology, functional membrane integrity, and survival capacity during cryopreservation of ram semen [127]. The co-supplementation of a Tris–citric-acid–egg-yolk–glycerol (3%)-based extender with trehalose (60 mM) and taxifolin hydrate (10 µM) shows improvement in sperm viability, mitochondrial activity, and the expression of GCLC, NQO1, and GSTP1gene along with significant reduction in oxidative stress, lipid peroxidation, and DNA damage during cryopreservation of ram semen [128]. The combined supplementation of trehalose (25 mmol/L) along with iodixanol (5%) into a Tris-based extender shows a synergistic effect with a significant improvement in sperm motility, longevity, and morphological and functional integrity of cryopreserved ram semen as shown in Table 4 [129].

Dithioerythritol: It displays a similar antioxidant effect to dithiothreitol and is identified as a protamine disulfide bond-breaking agent. Dithioerythritol (0.5, 1, 2 mM) has been supplemented to a basic extender during the cooled liquid storage of Merino ram sperm at 5 °C for 3 days, although no significant positive effect was observed on sperm motility indices and lipid peroxidation status after dithioerythritol treatment. However, GPx and GSH activities considerably improved with the best (2 mM) dithioerythritol treatment [71]. The addition of dithioerythritol (1 and 2 mM) to the semen-freezing extender conserved sperm motility and other CASA parameters but showed no significant effect on membrane integrity, acrosome integrity, mitochondrial activity, and other biochemical indices during cryopreservation of Merino ram semen as shown in Table 4 [130].

### 3.5. Fatty Acid and Oil Supplementations

The ovine sperm cell has a relatively higher PUFA content than other species, which defines the sensitivity of spermatozoa to freezing damage during cryopreservation. The elevated level of PUFAs within the sperm functional membrane is likely to affect their fluidity and fighting against cold shock due to the existence of several double bonds. The cryopreservation of Dorper ram semen led to changes in sperm energy metabolism and membrane integrity, with an increase in the number of differential metabolites and in cryo-damage during cryopreservation [131]. The supplementation of egg yolk enriched with different sources of PUFAs like linoleic acid, flaxseed oil, olive oil, and fish oil into a Tris-based preservation medium improved sperm motility/viability and membrane functionality, with reduced structural abnormalities during 4 °C storage of Afshari ram semen for 5 days [132]. The supplementation of oleic acid (0.5 and 1 mM) into a Tris-based preservation medium improved sperm motility parameters evaluated by CASA/viability, membrane functionality, SOD activity, and total antioxidant content, along with reduced nitric oxide and MDA levels during the liquid storage of Qezel ram semen for 3 days [133]. The supplementation of the ram diet with omega-3 and 6 fatty acid 35 g/ram/day improved the sperm quality parameters and testosterone concentration [134]. Supplementing docosahexaenoic acid (0.30 to 0.45 g per gram) to a semen-freezing extender during cryopreservation preserved the sperm motility parameters, membrane integrity, survival, and fertility rate of Moghani ram semen post-thaw [135]. The supplementation of PUFAs like palmitic acid, fish oil (2%) to a ram diet improves semen concentration with limited effect on sperm quality parameters like semen volume, CASA parameters, ability to penetrate artificial mucus, and lipid peroxidation during liquid storage of Belclare, Texel, and Suffolk ram semen at 4 °C as shown in Table 5 [136].

The supplementation of a basic ram diet with sunflower oil (linoleic acid 5%) and extra virgin olive oil (oleic acid 5%) harms sperm motility/viability/membrane integrity of post-thaw ram semen. Sperm VSL, VCL, VAP, and linearity of linoleic acid fed ram is not affected. Linoleic acid also does not affect the sex-sorting and cryo-survival ability of post-thaw ram sperm [137]. It might be due to excessive dietary lipid load and fatty acid imbalance. High linoleic acid from sunflower increases sperm membrane PUFA content, making it highly susceptible to testicular lipid peroxidation in the absence of proportional antioxidant supplementation. Simultaneously, the high total fat level disrupts rumen fermentation and induces systemic inflammation, leading to reduced testosterone and increased testicular oxidative stress. The supplementation of a basic ram diet with fish oil and palm oil and later semen preservation in a soya lecithin (SL)- and egg yolk (EY)-based extender show superior sperm motility/acrosome/membrane integrity, mitochondrial activity, morphological and apoptotic features with higher fertilizing ability post-thaw compared with palm oil-fed ram semen [138]. The supplementation of an omega-3 PUFA (0.4 mM) dose into Tris–egg-yolk-based semen-preservation medium shows higher sperm motility/viability/acrosome/membrane integrity during liquid storage at 4 °C for 48 h along with the cryopreservation of ram semen [139]. The supplementation of the basic ram diet with fish oil (2.5%) and vitamin C (300 mg/kg DM) has a positive synergistic effect on Moghani ram sperm motility indices, acrosome/membrane integrity, along with lactate dehydrogenase, docosahexaenoic acid, and possibly fertility rate [140]. Dietary inclusion of fish oil (3%) into the basic ram diet improves the semen fatty acid profile (DHA, linoleic acid, butyric acid, stearic acid, palmitic acid) and sperm quality parameters. Still, it has no significant effect on cholesterol concentration of post-thaw Criollo Araucano and Zandi ram semen [141,142]. The supplementation of alpha lipoic acid (0.1 mM) into a semen-preservation medium enhances sperm CASA indexes/membrane/acrosome integrity, T-AOC, CAT, SOD, MMP activity along with a significant decline in MDA and ROS content during the 4 °C preservation of Hu ram semen for 5 days as shown in Table 5 [26].

### 3.6. Supplementation of Nanoparticles for Ram Semen Preservation

The supplementation of curcumin nanoparticles (25 µM) into a Tris-based semen-preservation medium enhances sperm viability/motility indexes/membrane/acrosome integrity, antioxidant enzyme activity, fertility rate, along with a significant decline in ROS, MDA content, and early apoptotic changes in cryopreserved epididymal ram semen [143]. The supplementation of cerium oxide (CeO_2_) nanoparticles (220 µg/mL) into a soya-lecithin-based semen-preservation medium enhances sperm CASA parameters/membrane/acrosome integrity along with a significant decline in ROS production and DNA damage during 4 °C preservation of Sarda ram semen up to 96 h [144]. The supplementation of selenium nanoparticles (SeNps-1 µg/mL) into a Tris-based semen-freezing medium enhances sperm CASA parameters/viability/membrane integrity, does not affect oxidation status and PRDX5 gene expression, and also leads to a significant decline in DNA damage and morphological abnormalities during cryopreservation of Bafra ram semen [145]. The supplementation of cysteamine loaded on selenium nanoparticles (Cys-SeNps-1 µg/mL) into a Tris-based semen-freezing medium enhances sperm CASA parameters/viability/membrane/DNA integrity, SOD activity, along with a significant decline in MDA content and morphological abnormalities during cryopreservation of Sanjabi ram semen as shown in Table 6 [70].

Zinc oxide nanoparticle (1 µg/mL) addition into a Tris-based semen-freezing medium has a significant positive effect on sperm motility indexes/viability/membrane/DNA integrity, SOD activity, and total antioxidant capacity, along with a significant decline in MDA content and morphological abnormalities during the 4 °C preservation of ram caudal epididymal spermatozoa up to 72 h [146]. Another study revealed that zinc oxide nanoparticles (10–200 µg/mL) in a Tris-based semen-freezing medium have no significant effect on sperm CASA parameters, plasma membrane and acrosome integrity because of low bioavailability and dose-dependent inertness; however, they cause the significant improvement of sperm mitochondrial membrane potential during cryopreservation of Santa Inés ram semen [147]. The supplementation of copper oxide nanoparticles doped with zinc oxide nanoparticles and coated with quercetin 5 µg/mL into a Tris-based semen-freezing medium enhances sperm motility/viability/membrane/DNA integrity, along with a significant decline in MDA content and morphological abnormalities during the cryopreservation of Sanjabi ram semen, as shown in Table 6 [148].

### 3.7. Other Valuable Supplements Used for Ram Semen Preservation

Astaxanthin is a red keto-carotenoid pigment with confirmed antioxidant activity against oxidative damage and can prevent LPO by penetrating biological membranes as well as suppressing ROS-mediated damage to DNA, lipids, and proteins. Astaxanthin (3.5 µM) addition into a basic extender improves sperm CASA parameters, plasma/acrosome membrane integrity, viability, MMP and T-AOC, along with reduction in MDA and ROS content during 4 °C preservation of Hu ram semen for 5 days [20]. Astaxanthin (2 and 4 µM) addition into a basic extender improves sperm CASA parameters, plasma/acrosome membrane integrity, viability, along with reduction in MDA and ROS content during 4 °C preservation of Han ram semen for 3 days [149]. Astaxanthin (2 and 4 µM) addition into a freezing extender improves sperm CASA parameters, plasma membrane integrity, viability, and fertility rate, along with a reduction in MDA content and acrosomal abnormalities, and has no effects on SOD and GSH-PX activities during the cryopreservation of Moghani ram semen as shown in Table 7 [150].

Lycopene is an acyclic open-chain unsaturated carotenoid with a chemical formula of C_40_H_56_ and a molecular weight of 536.87. As a natural antioxidant, the antioxidant capacity of lycopene is ten times higher than that of β-carotene and α-tocopherol, respectively. Lycopene is present extensively in many fruits and vegetables such as tomato, watermelon, pink guava, and pink grapefruit. Lycopene (2 µM) addition into a freezing extender improves sperm post-thaw motility parameters, structural/plasma/acrosome membrane integrity, viability, MMP, SOD, T-AOC, cleavage, and fertility rate, along with a reduction in MDA and ROS content during the cryopreservation of Oura-type Tibetan ram semen [151]. Lycopene (2.5 µM) addition into a basic extender improves sperm CASA parameters, viability, plasma/acrosome membrane integrity, MMP, SOD, CAT, T-AOC, along with reduction in MDA and ROS content during the 4 °C preservation of Hu ram semen for 5 days [18]. The supplementation of lycopene (3 mg/100 mL) to a Tris–egg yolk (TEY)-based extender shows significant improvement in sperm quality parameters with reduced structural abnormality in post-thaw Sapudi ram semen [50]. The supplementation of lycopene (0.5 or 2 mM) to a Tris-based extender shows significant improvement in sperm quality parameters like motility and viability with reduced oxidative stress damage during 5 °C Merino ram semen storage for 72 h as shown in Table 7 [69].

Quercetin is a flavonoid obtained from plants and vegetables with strong antioxidant properties due to the presence of three hydroxyl groups. The supplementation of quercetin (5 µg/mL) to a Tris–glycerol-based semen-freezing extender shows a significant improvement in sperm motion and kinetic parameters, viability, plasma membrane/acrosome integrity, and MMP during cryopreservation of Santa Inés ram semen [152]. Another study revealed quercetin (5 µg/mL) addition to a Tris–glycerol-based semen-freezing extender shows a significant improvement in sperm CASA parameters, plasma membrane/acrosome integrity, and reduced lipid peroxidation, such as MDA content, of post-thaw crossbred ram semen [15]. Another study on Lohi sheep confirmed the synergistic effect of exogenous adenosine triphosphate (1 mM) and quercetin (10 or 20 µM) supplemented individually or in combination on post-thaw sperm CASA parameters, viability, membrane integrity, and fertility rate using laparoscopic artificial insemination techniques as shown in Table 7 [153].

Idebenone: The supplementation of idebenone (10 µM) to a Tris-based semen-freezing extender shows a significant improvement in sperm motion and kinetic parameters, viability, plasma membrane/acrosome integrity, along with reduction in MDA level, which is an end product of lipid peroxidation during cryopreservation of ram semen [154]. Another study revealed that idebenone (4 µM) addition to a Tris-based semen extender shows significant improvement in sperm CASA parameters, viability, plasma membrane/acrosome integrity, total antioxidant capacity, SOD level, along with reduced peroxidative (MDA) content and nitrosative (nitric oxide) stress during the 4 °C storage of ram semen for 72 h [155]. Idebenone (4 and 8 µM) treatment of a basic extender alleviates the deltamethrin-mediated toxicity of ram semen and shows a significant recovery of sperm motility indexes, viability, membrane integrity, total antioxidant content, SOD activity, along with a significant decline of nitrate/nitrite and MDA content of Qezel ram semen during 4 °C storage for 72 h [156]. Idebenone (10 µM) treatment of a basic freezing extender shows a significant improvement in sperm CASA parameters, viability, plasma membrane/acrosome integrity, with reduced oxidative stress and DNA fragmentation during the conventional freezing method as shown in Table 7 [157].

Resveratrol: Resveratrol (50 or 75 µM) addition into a freezing extender improves sperm post-thaw motility parameters, plasma/acrosome/mitochondrial membrane integrity potential, CAT, ATP, SOD, SIRT1, GSH, GPx, and AMPK phosphorylation level, along with a significant reduction in MDA and DNA oxidative damage during the cryopreservation of small-tailed Han ram semen [158]. Resveratrol (200 or 400 µM) addition into a Triladyl-based freezing extender improves sperm post-thaw motility and biokinetic parameters, structural morphology, SOD, GSH activity, in vitro fertility rate in terms of cleavage and blastocyst rate, along with a significant reduction in MDA content and oxidative stress damage during the 5 °C preservation of Najdi ram semen up to 7 days [159]. Another study reported that resveratrol alone or resveratrol-loaded cyclodextrin (10 or 20 µg/mL) addition into a freezing extender improves post-thaw Kivircik ram sperm motility parameters assessed through CASA, plasma/acrosome/mitochondrial membrane integrity potential and capacitation status along with reduced oxidative stress damages during the cryopreservation of semen [160]. Resveratrol (50 µM) and antifreeze protein (AFP) type 1 (0.1 µg/mL) supplemented in combination into a semen-freezing medium do not show a synergic effect and have no impact on sperm kinematic parameters, capacitation status, membrane integrity, and mitochondrial activity during cryopreservation of ram semen. Resveratrol acts as an antioxidant targeting ROS-mediated mitochondrial damage, while AFP-I acts as a physical cryoprotectant targeting ice recrystallization. In ram semen, physical damage during freeze–thaw is the dominant cause of motility and membrane loss, so antioxidant protection alone is insufficient to improve CASA parameters. Thus, the lack of effect might reflects mechanistic mismatch and suboptimal dosing rather than true incompatibility [161]. Another study demonstrated that the addition of resveratrol to a semen-freezing medium could elevate the expression levels of dihydrolipoamide dehydrogenase (DLD), which is an energy-related protein, and oxidative stress-related (NDUFB9) proteins to improve the vitality of cryopreserved Mongolian sheep sperm as shown in Table 7 [162].

Melatonin: Melatonin (0.05, 0.1 and 0.2 mM) treatment of a basic extender shows significant improvement of sperm motility, plasma membrane/acrosome integrity, MMP, and total antioxidant content along with a significant decline in MDA content of ram semen during 4 °C storage for 72 h [163]. Melatonin (1 mM) treatment of a basic extender shows a significant improvement in sperm motility, viability, membrane integrity, and total antioxidant content along with a significant decline in abnormalities and MDA content during the 4 °C storage of Magra ram semen for 72 h [164]. Melatonin (15 and 60 µg/mL) treatment of a basic extender shows a significant improvement in sperm motility/viability/membrane/DNA integrity, SOD, and total antioxidant content along with a significant decline in morphological abnormalities and MDA content during the 4 °C storage of post-mortem recovered epididymal ram spermatozoa for 72 h [165]. The supplementation of melatonin (100 pm) to a Tris-based semen-freezing extender shows a significant improvement in sperm motion and kinetic parameters, viability, capacitation status, cleavage, in vitro fertility rate, and phosphatidylserine transition along with a reduction in oxidative stress during cryopreservation of ram semen in non-breeding season [166]. Exogenous melatonin implants improve sperm quality, DNA integrity, and mitochondrial activity, and mitigate the adverse effects of summer heat stress and oxidation during the non-reproductive season of crossbred Hu and Merino rams. It reduces abnormal sperm count through the regulation of the endocrine hormonal profile of seminal plasma with activation of the tryptophan metabolic pathway to protect the semen microenvironment [167,168]. Melatonin (10^-7^ M) addition into a freezing extender preserves the sperm mitochondrial membrane potential, membrane integrity, cytochrome c release from the mitochondrial matrix, and fertilization ability by inhibiting the mitochondrial permeability transition pore (mPTP) by acting on the MT1 site through the activation of the PI3K-Akt and GSK-3β pathways during the cryopreservation of ram semen [169]. During semen production, melatonin increases blood flow in the ram testicle and epididymis, and exerts a positive effect on spermatogenesis, seminal quality, capacitation status, chemotaxis, and fertilization through interaction with MT1 and MT2 receptors as shown in Table 7 [170].

**Table 7:** Effects of some other valuable non-enzymatic antioxidant supplementation on ram sperm quality parameters during liquid storage or cryopreservation.

Antioxidants	Optimal Conc.	Temp	Time	Ram Breed	Results	Ref.
Astaxanthin	3.5 µM	4 °C	5 days	Hu	Improved sperm CASA parameters, PMI, ACI, viability, MMP, and TAC with reduced MDA and ROS content.	[20]
Astaxanthin	2, 4 µM	4 °C	3 days	Han	Improved sperm CASA parameters, PMI, ACI, viability, with reduced MDA and ROS content.	[149]
Astaxanthin	2, 4 µM	−196 °C	Post-thaw	Moghani	Improved sperm CASA parameters, PMI, viability, and fertility rate with reduced MDA and acrosomal abnormalities and having no effects on SOD and GPx activities.	[150]
Lycopene	2 µM	−196 °C	Post-thaw	Oura-type Tibetan	Improved sperm motility parameters, PMI, ACI, structural integrity, viability, MMP, SOD, TAC, cleavage, and fertility rate with reduced MDA and ROS content.	[151]
Lycopene	2.5 µM	4 °C	5 days	Hu	Improved sperm CASA parameters, viability, PMI, ACI, MMP, SOD, CAT, TAC with reduced MDA and ROS content.	[18]
Lycopene	3 mg/100 mL	−196 °C	Post-thaw	Sapudi	Improved sperm quality parameters with reduced structural abnormality.	[50]
Lycopene	0.5, 2 mM	5 °C	72 h	Merino	Improved sperm quality parameters such as motility and viability while reduced oxidative stress-related damage.	[69]
Quercetin	5 µg/mL	−196 °C	Post-thaw	Santa Ines	Improved sperm motion and kinetic parameters, viability, plasma membrane/acrosome integrity, and MMP.	[152]
Quercetin	5 µg/mL	−196 °C	Post-thaw	Cross bred	Improved sperm CASA parameters, PMI, and ACI with reduced LPO-like MDA content.	[15]
Adenosine triphosphate & Quercetin	1 mM 10, 20 µM	−196 °C	Post-thaw	Lohi	Beneficial synergistic effect on sperm CASA parameters, viability, membrane integrity, and fertility rate in laparoscopic AI.	[153]
Idebenone	10 µM	−196 °C	Post-thaw	Crossbred	Improved sperm motility and kinetic parameters, viability, PMI, and ACI, with reduced MDA levels.	[154]
Idebenone	4 µM	4 °C	72 h	N/A	Improved sperm CASA parameters, viability, PMI, ACI, TAC, and SOD level with reduced MDA and nitrosative (NO) stress.	[155]
Idebenone	4, 8 µM	4 °C	72 h	Qezel	Shows significant recovery of sperm motility indexes, viability, PMI, TAC, and SOD activity with reduced nitrate/nitrite and MDA content.	[156]
Idebenone	10 µM	−196 °C	Post-thaw	N/A	Improved sperm CASA parameters, viability, PMI, and ACI with reduced oxidative stress and DNA fragmentation.	[157]
Resveratrol	50, 75 µM	−196 °C	Post-thaw	Han	Improved sperm CASA parameters, PMI, ACI, MMP, CAT, ATP, SOD, SIRT1, GSH, GPx, and AMPK phosphorylation with reduced MDA and DNA damage.	[158]
Resveratrol	200, 400 µM	5 °C	7 days	Najdi	Improved sperm CASA parameters, structural morphology, SOD, GSH activity, and fertility rate with reduced MDA content and oxidative stress damage.	[159]
Resveratrol alone or RES loaded cyclodextrin	10, 20 µg/mL	−196 °C	Post-thaw	Kivircik	Improved sperm CASA parameters, PMI, ACI, MMP, and capacitation status with reduced oxidative stress damages.	[160]
Resveratrol and antifreeze protein type-1	50 µM 0.1 µg/mL	−196 °C	Post-thaw	N/A	Does not show synergic effect, having no impact on sperm kinematic parameters, capacitation status, membrane integrity, and mitochondrial activity.	[161]
Melatonin	0.05–0.2 mM	4 °C	72 h	N/A	Improved sperm motility, PMI, ACI, MMP, and TAC with a significant decline in MDA content.	[163]
Melatonin	1 mM	4 °C	72 h	Magra	Improved sperm motility, viability, PMI, and TAC with a significant decline in structural abnormalities and MDA content.	[164]
Melatonin	15, 60 µg/mL	4 °C	72 h	N/A	Improved sperm motility/viability, PMI, DNA integrity, SOD, and TAC with a significant decline in morphological abnormalities and MDA content.	[165]
Melatonin	100 pm	−196 °C	Post-thaw	N/A	Improved sperm CASA indexes, viability, capacitation status, fertility rate, and phosphatidylserine transition with reduced oxidative stress.	[166]
Melatonin implants	Exogeneous	−196 °C	Post-thaw	Hu & Merino	Improved sperm quality, DNA integrity, and MMP by reducing oxidation due to summer heat stress, abnormality, and activating the tryptone metabolic pathway during the non-reproductive season.	[167,168]
Melatonin	10^−7^ M	−196 °C	Post-thaw	N/A	Improved sperm MMP, PMI, cytochrome C release from the mitochondrial matrix, and fertility by inhibiting mitochondrial permeability transition pore (mPTP) by acting on the MT1 site through the activation of the PI3K/Akt and GSK-3β pathways.	[169]
Crocin	0.5, 1 mM	5 °C	72 h	Chios	Improved sperm motility and kinematic parameters, viability, intracellular GSH level, and HSP expression with a significant decline in sperm apoptosis rate.	[171]
β-carotene	20 mg/L	−196 °C	Post-thaw	Hu	Improved sperm CASA indexes, PMI, ACI, TAC, CAT, SOD, and MMP activity with a significant decline in MDA, ROS content, apoptosis rate, and cytochrome-C release.	[172]

Note: CASA: Computer-assisted sperm analysis; CAT: Catalase; PMI: Plasma membrane integrity; ROS: Reactive oxygen species; LPO: Lipid peroxidation; ACI: Acrosomal integrity; GPx: Glutathione peroxidase; GSH: Reduced glutathione; SOD: Superoxide dismutase; CAT: Catalase; SIRT1: Sirtuin-1; ATP: Adenosine triphosphate; TAC: Total antioxidant contents; MMP: Mitochondrial membrane potential; MDA: Malondialdehyde. AMPK: Adenosine monophosphate-activated protein kinase; GSK-3β: Glycogen synthase kinase-3 beta; MT1: Melatonin receptor-1; PI3K/Akt: Phosphoinositide 3-kinase pathway.

The supplementation of crocin (0.5, 1 mM) into a soya lecithin-based semen-preservation medium enhances sperm motility and kinematic parameters, viability, intracellular glutathione level, and heat shock protein expression along with a significant decline in sperm apoptosis rate during 5 °C preservation of Chios ram semen for 3 days [171]. The supplementation of β-carotene (20 mg/L) into a Tris-based semen-preservation medium enhances sperm CASA indexes/membrane/acrosome integrity, T-AOC, CAT, SOD, and MMP activity, along with a significant decline in MDA, ROS content, apoptosis rate, and cytochrome-c released during cryopreservation of Hu ram semen as shown in Table 7 [172].

Based on current evidence, vitamins (vitamin E and C) and amino acids represent the best-supported non-enzymatic antioxidants for ram semen cryopreservation. Melatonin; resveratrol; quercetin; and sugars—such as fructose, sucrose, and trehalose—show moderate evidence and are recommended for inclusion. Carotenoids such as β-carotene, astaxanthin, and lycopene, and mitochondrial-targeted compounds like idebenone, are mechanistically promising but remain experimental due to limited ram-specific trials. Plant-, fruit-, herb-, and vegetable-based extracts and other polyphenolic compounds also remain experimental due to limited studies and high variability, and require standardization before recommendation. Fatty acid and oil supplementation represents a diet-based non-enzymatic antioxidant strategy for ram semen preservation. Compared to vitamins and thiols that act directly in the extender, fatty acids require 4–6 weeks of dietary feeding to incorporate into the sperm membrane and improve sperm quality. Therefore, fatty acid supplementation is conditionally recommended as an adjunct, but cannot replace extender-based antioxidants. The direct addition of oils to semen extenders is not recommended due to toxicity and a lack of sufficient data. Nanoparticle-based antioxidants represent a novel but unvalidated approach and cannot be recommended until toxicity is assessed. Future research should prioritize mechanism-based studies on various classes of non-enzymatic antioxidants and evaluate their interactions with standard cryoprotectants.

## 4. Conclusions

Ram semen preservation, whether by liquid storage at low temperature or by cryopreservation at −196 °C, remains a critical step because the quality of preserved sperm strongly determines the success of artificial insemination. Despite significant improvement in the development of various semen extenders for different storage temperatures, along with the addition of different exogenous supplements, the preservation process can still cause sub-lethal damage to ram spermatozoa, including reduced motility indexes, loss of membrane integrity, DNA damage, and altered protein functions with loss of energy metabolism and antioxidant enzyme activity. The present review emphasizes the oxidative stress damage that ram spermatozoa experience during the liquid storage of semen or the cryopreservation process, along with the efficacy of supplementing various exogenous non-enzymatic antioxidants as oral feeding in the ram diet or in vitro addition into the preservation medium. The supplementation of basic extenders with different non-enzymatic antioxidant substances—particularly protein and amino acids, plant extracts, vitamins, carotenoids, phenolic compounds, sugars, fatty acids, seminal plasma, and nanoparticles—can improve the ram sperm motility indices, viability, structural morphology, DNA integrity, mitochondrial function, antioxidant enzyme activity, and overall antioxidant capacity during ram semen liquid storage and cryopreservation. In contrast, the supplementation of these different non-enzymatic antioxidant compounds to a semen extender considerably reduced highly detrimental reactive oxygen species (ROS), malondialdehyde (MDA) production, sperm morphological abnormalities, apoptosis rate, and the production of other free radicals and non-radicals during preservation.

The optimal dose and efficacy of non-enzymatic antioxidant supplements in semen-preservation media are influenced by several factors. These include storage temperature and duration, cryopreservation technique, extender type, antioxidant dissolution medium, equilibration time, and cryoprotectant used. These beneficial effects of non-enzymatic antioxidant supplementation eventually led to improved sperm quality characteristics and a higher fertility rate after insemination. However, further studies are required to elucidate the exact mechanisms of action of these non-enzymatic antioxidant supplements and their interactions with various cryoprotectants. Additionally, the activation of specific cryoprotective marker genes during the liquid storage or cryopreservation of ram semen needs to be investigated.

## Figures and Tables

**Table 1:** Effects of different vitamins or vitamin-like compound supplementation on ram sperm quality parameters during liquid storage or cryopreservation.

Antioxidants	Optimal Conc.	Temp	Time	Ram Breed	Results	Ref.
Vitamin E	1 mg/mL	5 °C	72 h	Kail	Increased sperm motility, LIN, STR, BCF. No effect on viability, PMI.	[23]
Vitamin E-Loaded Cyclodextrin		−196 °C	Post-thaw	N/A	Improved sperm T.M, P.M, LIN, VSL, VAP, VCL, and PMI. Decreased lipid peroxidation.	[47]
Vit E (Trolox)	50 µM	−196 °C	Post-thaw	Santa Ines	Significantly reduced LPO and H_2_O_2._	[14]
Vit E (Trolox)	60, 120 µM	−196 °C	Post-thaw	N/A	Improved sperm CASA parameters like P.M, VSL, VAP, STR, LIN, and other indexes like PMI, ACI, MMP.	[48]
Vit E (Trolox)	1, 5 mM	5 °C, 15 °C	96 h	N/A	Extender-based effect; Improved acrosomal status with reduced sperm motility, viability, and mitochondrial activity.	[49]
Ascorbic acid/Vitamin C	3 mg/100 mL	−196 °C	Post-thaw	Sapudi	Improved sperm motility, viability, and plasma membrane integrity with reduced sperm abnormality	[50]
Ascorbic acid/Vitamin C	0.5 mM	−196 °C	Post-thaw	Noemi	Improved sperm T.M, P.M, VSL, VCL, VAP, STR, LIN, viability, PMI, and ACI with reduced sperm abnormality.	[51]
Ascorbic acid/Vitamin C	4.5 mg/mL	4 °C	72 h	N/A	Improved sperm acrosome integrity. No effect on viability and morphology, Lower sperm motility and membrane integrity.	[52]
Vitamin B_12_	2 mg/mL	−196 °C	Post-thaw	Dallagh	Improved motility indexes, viability, and normality of spermatozoa.	[53]
Vitamin B_12_	1–2 mg/mL	−196 °C	Post-thaw	Arabi	Improved sperm motility, viability, membrane integrity, morphology, TAC, CAT, GPx activity. Reduced MDA content.	[54]
Vitamin D	50 ng/mL	−196 °C	Post-thaw	Merino	Improved sperm motility indexes, PMI, ACI, MMP. Reduced DNA damage.	[55]
Coenzyme Q_10_	50 μmol/L	Room-Temp	5 Days	Hu	Improved sperm P.M, VAP, VCL, ALH, PMI, MMP, ATP, SOD, CAT, TAC. Reduced ROS and MDA levels.	[21]
Coenzyme Q_10_	2 µM	−196 °C	Post-thaw	N/A	Improved sperm motility, viability, PMI, ACI, MMP. Reduced MDA level, apoptotic-like changes, abnormal morphology.	[56]
Myo-inositol	7.5 mM	−196 °C	Post-thaw	Mehraban	Improved sperm T.M, P.M, VCL, VAP, viability, PMI, TAC, MMP. Reduced ROS level and LPO.	[57]

Note: T.M: total motility; P.M: Progressive motility; VSL: Straight line velocity; VCL: Curvilinear velocity; VAP: Average path velocity; STR: Straightness; LIN: Linearity; ALH: Amplitude of latera head displacement; CAT: Catalase; LIN: Linearity of sperm; STR: Straightness; PMI: Plasma membrane integrity; BCF: Beat cross frequency; SOD: Superoxide dismutase; LPO: Lipid peroxidation; ACI: Acrosomal integrity; GPx: Glutathione peroxidase; H_2_O_2_: Hydrogen peroxide; TAC: Total antioxidant contents; ATP: Adenosine triphosphate; ROS: Reactive oxygen species; MMP: Mitochondrial membrane potential; MDA: Malondialdehyde.

**Table 3:** Effects of different plant-, fruit-, herb-, and vegetable-based extract and polyphenolic compound supplementation on ram sperm quality parameters during liquid storage or cryopreservation.

Antioxidants	Optimal Conc.	Temp	Time	Ram Breed	Results	Ref.
Rosemary extract	4, 6%	−196 °C	Post-thaw	Chal	Improved sperm CASA parameters, viability, and PMI, having no effect on acrosome and capacitation status with reduced MDA damage.	[88]
Rosemary honey	5%	−196 °C	Post-thaw	Rasa Aragonesa	Improved sperm motility, viability, membrane integrity, and embryo development rate with reduced structural abnormality.	[89]
Rosemary essential oil	12.5, 25 μg/mL	−196 °C	Post-thaw	N/A	Improved sperm CASA parameters, viability, PMI, and MMP with reduced oxidative stress damage.	[90]
Gallic acids Carnosic acids	2 mM 0.5 mM	−196 °C	Post-thaw	Merino	Improved sperm CASA parameters, PMI, ACI, and mitochondrial membrane potential.	[91]
Moringa leaf extract	0.64 mg/mL	−196 °C	Post-thaw	Awassi	Improved sperm motility, viability, PMI, ACI, SOD, LDH, and GPx activity with reduced total cholesterol, LDL, MDA, NO, and copper content.	[92]
Moringa leaf extract	40 mg/kg in diet	−196 °C	Fresh & post-thaw	Barki	Improved semen volume, sperm concentration, sperm motility/viability/membrane integrity, TAC, SOD, CAT, GPx, G.R, ALP, ACP, and ascorbic acid with reduced DNA damage and MDA content.	[93]
Moringa seed extract	5, 10 mg/mL	−196 °C	Post-thaw	Hair	Improved sperm motility, viability, PMI, ACI, mitochondrial/antimicrobial/antioxidant activity with higher IVF rate.	[94,95]
Quinoa seed extract	750–1000 µg/mL	−196 °C	Post-thaw	Rahmani	Improved sperm motility, viability, ACI, SOD1, TAC, CAT, GABPB1, and GPX1 gene expression with significant decline in apoptotic changes, H_2_O_2_, MDA content, and CASP3 gene expression.	[96]
Cinnamon extract	100 µL/mL	4 °C	72 h	Arabi	Improved sperm CASA parameters, viability, acrosome integrity, and normal morphology.	[97]
Opuntia ficus indica cladodes extract	1%	5 °C	72 h	N/A	Improved sperm motility, viability, PMI, and ACI with reduced lipid peroxidation and DNA damage.	[98]
Entada abyssinica bark extract	375 µg/mL	−196 °C	Post-thaw	Ossimi	Improved sperm motility, viability, PMI, ACI, and TAC having no significant effect on ALT, AST, and LDH activity, with a significant decline in sperm necrotic and apoptotic changes.	[99]
Lavender extract	150 µg/dL	−196 °C	Post-thaw	Shall	Improved sperm motility, viability, PMI, ACI, MMP, SOD, and fertility, while having no effect on GPx activity, with reduced apoptotic changes, H_2_O_2_, MDA, and DNA damage.	[100]
Ginger and echinacea extract	10 & 20 mg/L	−196 °C	Post-thaw	N/A	Improved sperm motility, viability, ACI, PMI, mitochondrial activity, and fertility parameters with reduced lipid peroxidation and DNA damage.	[101]
Terminalia chebula & Thymbra spicata extract	3000 µg/mL	37 °C	Fresh	N/A	Improved sperm viability and DNA integrity with a significant decline in LPO and MDA content under normal and H_2_O_2-_induced OS.	[102]
Curcumin	20 µmol/L	4 °C		Hu	Improved sperm viability, motility indexes, PMI, ACI, TAC, CAT, SOD activity, and metabolic profile with a significant decline in LPO, MDA, and ROS content.	[103]
Clove bud extract	35, 75 µg/mL	−196 °C	Post-thaw	N/A	Improved sperm viability, motility, membrane integrity, and antioxidant activity.	[104]
Fennel extract	10 mg/L	−196 °C	Post-thaw	Ghezel	Improved sperm viability, motility indexes, membrane integrity, and mitochondrial activity with significantly reduced LPO, apoptotic changes, and abnormality.	[105]
Tinospora cordifolia	1 g/kg B.W in diet		6 months	Muzaffarnagari	No considerable effect on physiological, biochemical, seminal, and testosterone conc of semen; however, improved SOD, CAT, and cholesterol concentration.	[106]
Oregonin	100 µM	5 °C	48 h	Local bred	Improved sperm motility parameters evaluated by CASA, morphological parameters, mitochondrial activity, and fertilization potential.	[107]
Cactus seed oil	1 or 2%	5 °C	72 h	Boujaad	Improved sperm motility, viability, and morphological parameters along with reduced LPO and DNA fragmentation.	[108]
Punicalagin (pomegranate extract)	30 µM	4 °C	5 days	Hu	Improved sperm CASA indexes, PMI, ACI, TAC, CAT, SOD, and MMP activity with significant decline in MDA and ROS content.	[24]
Chlorogenic acid	0.8 mg/mL	4 °C	5 days	Hu	Improved sperm viability, CASA indexes, PMI, ACI, TAC, ATP, CAT, SOD activity with significant decline in MDA and ROS content.	[22]
Punicalagin (pomegranate extract)	15 µM	−196 °C	Post-thaw	Hu	Improved sperm CASA indexes, PMI, ACI, TAC, ATP, MMP, CAT, SOD, GSH activity and Nrf2/PGC-1α pathway with significant decline in apoptosis rate, MDA and ROS content.	[109]
Puerarin	75 µM	−196 °C	Post-thaw	Hu	Improved sperm CASA indexes, PMI, ACI, TAC, CAT, SOD, MMP, and in vitro fertility rate with significant decline in MDA and ROS content.	[25]
Silymarin Caproic acid	100 µg/mL 0.312%	5 °C	72 h	N/A	Improved sperm viability, motility indexes, PMI, ACI with significant decline in MDA content.	[110]
Ellagic acid Ebselen	1, 2 mM 10–40 µM	5 °C	72 h	Merino	Improved sperm viability, motility indexes, MMP, DNA integrity, antioxidant potential and GSH content with significant decline in LPO.	[111]
Quinic acid	100 µg/mL	−196 °C	Post-thaw	Ramlic	Improved sperm CASA indexes, viability, MMP, DNA integrity, TAC with significant decline in MDA, ROS content and redox parameters.	[112]
Spirulina platensis & Salvia verbenaca extract	1.25 µg/mL 3.75, 6.25 μg/mL	4 °C	24 h	N/A	Improved sperm motility indexes, viability, PMI and fertility with significant decline in LPO and sperm abnormality.	[113]

Note: CASA: Computer-assisted sperm analysis; CAT: Catalase; PMI: Plasma membrane integrity; SOD: Superoxide dismutase; LPO: Lipid peroxidation; ACI: Acrosomal integrity; GPx: Glutathione peroxidase; H_2_O_2_: Hydrogen peroxide; TAC: Total antioxidant contents; LDH: Lactate dehydrogenase; NO: Nitric oxide; GSH: Reduced glutathione; LDL: Low density lipoprotein; G.R: Glutathione reductase; ALP: Alkaline phosphatase; ACP: Acid phosphatase; GABPB1: GA Binding Protein Transcription Factor Subunit Beta-1; ATP: Adenosine triphosphate; ROS: Reactive oxygen species; MMP: Mitochondrial membrane potential; MDA: Malondialdehyde; CASP3: cysteine-aspartic acid protease; IVF: In vitro fertilization; ALT: Alanine aminotransferase; AST: Aspartate aminotransferase.

**Table 4:** Effects of supplementation with different sugars or sugar-like compounds on ram sperm quality parameters during liquid storage or cryopreservation.

Antioxidants	Optimal Conc.	Temp	Time	Ram Breed	Results	Ref.
Sucrose, trehalose or raffinose	(50–100 mM)	−196 °C	Post-thaw	Moghani	Improved sperm motility parameters, viability, ACI, PMI, and fertility rate with reduced acrosomal and other abnormalities.	[114]
Sucrose	0.4–0.8 M	−196 °C	Post-thaw	Merino	Improved sperm motility, morphology, membrane integrity, DNA integrity, and cryo-survival.	[115]
Trehalose, sucrose, raffinose with Vit E	100 mM 60 mM 10 mM 2 mM	−196 °C	Post-thaw	Afshari	Improved sperm quality parameters like viability and total/progressive motility during cryopreservation.	[116]
Glycerol, trehalose or sucrose	Mixing at cooling from 30 to 5 °C	−196 °C	Post-thaw	Australian Merino	Improved sperm motility and plasma membrane/acrosome integrity during cryopreservation.	[117]
Trehalose	50 mM	5 °C	30 h	Chios	Improved sperm motility, viability, and membrane integrity with a significant decline in sperm abnormalities.	[74]
Trehalose, sucrose or glucose sugars	30–210 mM	−196 °C	Post-thaw	N/A	Improved sperm motility, viability, acrosome integrity, and in vivo fertility.	[118]
Trehalose	100 mOsm	−196 °C	Post-thaw	Pampinta	Improved sperm motility, plasma membrane/acrosome integrity, and in vivo fertility.	[119]
Trehalose Dimethylacetamide	100 mOsm 3 or 6%	−196 °C	Post-thaw	Santa Ines	Reduced sperm motility indexes; however, have a Positive effect on PMI and acrosomal integrity rate.	[120]
Glycerol Trehalose	3% 60 mM	−196 °C	Post-thaw	Merino	Improved sperm motility, viability, acrosome integrity, and mitochondrial activity.	[121]
Trehalose		−196 °C	Post-thaw	N/A	Modified the proteomic profile to show antioxidation, glycolysis, and cryotolerance to various stresses.	[122]
Trehalose with LDL	100 mM 8%	−196 °C	Post-thaw	Crioulo	Improved sperm motility parameters, PMI, and ACI during cryopreservation.	[123]
Trehalose and Glycerin	100 mM 5%	−196 °C	Post-thaw	Zandi	Improved sperm CASA parameters, PMI, capacitation status, and MMP with a significant decline in apoptosis rate, abnormality, and MDA content.	[124]
Trehalose and EDTA		−196 °C	Post-thaw	N/A	Beneficial synergistic effect on sperm motility, ACI, and cryo-survival probably by removing calcium from the medium.	[125]
Trehalose	100 mM	−196 °C	Post-thaw	Akkaraman	Improved sperm motility, morphology, viability, CAT activity, and PMI with no effect on MDA content, GSH, and GPx activity.	[66,126]
Protease inhibitor antipain with Trehalose	10 µM 30, 60 mM	−196 °C	Post-thaw	N/A	Improved total and progressive motility of sperm, morphology, PMI, and survival capacity.	[127]
Trehalose and taxifolin hydrate	60 mM 10 µM	−196 °C	Post-thaw	N/A	Improved sperm viability, MMP, and expression of GCLC, NQO1, and GSTP1gene with significant decline of OS, LPO, and DNA damage.	[128]
Trehalose with iodixanol	25 mmol/L 5%	−196 °C	Post-thaw	N/A	Synergistic effect with significant improvement in sperm motility, longevity, and morphological and functional membrane integrity.	[129]
Dithioerythritol	2 mM	5 °C	72 h	Merino	No significant effect on sperm motility and lipid peroxidation with higher GPx and GSH activity.	[71]
Dithioerythritol	1, 2 mM	−196 °C	Post-thaw	Merino	Improved sperm T.M, P.M, and other CASA parameters. No effect on PMI, ACI, mitochondrial activity and other biochemical parameters.	[130]

Note: CASA: Computer-assisted sperm analysis; CAT: Catalase; PMI: Plasma membrane integrity; OS: Oxidative stress; LPO: Lipid peroxidation; ACI: Acrosomal integrity; GPx: Glutathione peroxidase; GSH: Reduced glutathione; GCLC: Glutamate-cysteine ligase catalytic subunit; NQO1: NADH quinone dehydrogenase-1; GSTP1: Glutathione S-transferase Pi-1; MMP: Mitochondrial membrane potential; MDA: Malondialdehyde.

**Table 5:** Effects of supplementation with different fatty acids and oils on ram sperm quality parameters during liquid storage or cryopreservation.

Antioxidants	Optimal Conc.	Temp	Time	Ram Breed	Results	Ref.
Oleic acid	0.5, 1 mM	4 °C	3 days	Qezel	Improved sperm motility/CASA parameters, viability, PMI, SOD, and TAC content with reduced nitric oxide and MDA levels.	[133]
Omega 3 and 6 fatty acids	35 g/ram/day in diet for 10 weeks	** **	Fresh	Shall	Improved volume, sperm concentration, motility, PMI, morphology, DNA integrity, and testosterone concentration.	[134]
Docosahexaenoic acid (DHA)	0.30, 0.45 g	−196 °C	Post-thaw	Moghani	Improved sperm progressive motility, viability, membrane integrity, and fertility rate.	[135]
Palmitic acid & fish oil	2% of diet for 9 weeks	4 °C	72 h	Belclare, Texel & Suffolk	Improved sperm concentration with limited effect on semen volume, CASA parameters, ability to penetrate artificial mucus, and LPO.	[136]
Linoleic acid & Oleic acid	Each 5% of diet for 6 weeks	−196 °C	Post-thaw	N/A	Negative impact on sperm CASA parameters, viability, and ACI, with no effect on sex-sorting and cryo-survival ability.	[137]
Fish oil Palm oil	Dietary supplementation	−196 °C	Post-thaw	N/A	Improved sperm motility, ACI, PMI, mitochondrial activity, and morphological and apoptotic features with higher fertilizing ability.	[138]
Omega-3 PUFAs	0.4 mM	4 °C & −196 °C	48 h & post-thaw	N/A	Improved sperm motility, viability, and acrosome/plasma membrane integrity.	[139]
Fish oil and vitamin C	2.5% of diet 300 mg/kg for 14 weeks		After 14 weeks semen	Moghani	Positive synergistic effect on sperm motility indexes, acrosome/membrane integrity with LDH, DHA, and fertility rate.	[140]
Fish oil	3% of diet for 8 & 13 weeks	−196 °C	Post-thaw	Criollo Araucano & Zandi	Improved semen fatty acid profile (DHA, linoleic acid, butyric acid, stearic acid, palmitic acid) and sperm quality parameters but do not affect cholesterol concentration.	[141,142]
Alpha lipoic acid	0.1 mM	4 °C	5 days	Hu	Improved sperm CASA indexes, PMI, ACI, TAC, ATP, SOD, and MMP activity with a significant decline in MDA and ROS content.	[26]

Note: CASA: Computer-assisted sperm analysis; TAC: Total antioxidant contents; PMI: Plasma membrane integrity; SOD: Superoxide dismutase; LPO: Lipid peroxidation; ACI: Acrosomal integrity; LDH: Lactate dehydrogenase; DHA: Docosahexaenoic acid; ATP: Adenosine triphosphate; ROS: Reactive oxygen species; MMP: Mitochondrial membrane potential; MDA: Malondialdehyde.

**Table 6:** Effects of the supplementation of different nanoparticles on ram sperm quality parameters during liquid storage or cryopreservation.

Antioxidants	Optimal Conc.	Temp	Time	Ram Breed	Results	Ref.
Curcumin nanoparticles	25 µM	−196 °C	Post-thaw	N/A	Improved sperm viability, motility indices, PMI, ACI, antioxidant enzyme activity, and fertility rate with a significant decline in ROS, MDA content, and early apoptotic changes.	[143]
Cerium oxide (CeO_2_) nanoparticles	220 µg/mL	4 °C	96 h	Sarda	Improved sperm CASA parameters, PMI, and ACI with a significant decline in ROS production and DNA damage.	[144]
Selenium nanoparticles (SeNps)	1 µg/mL	−196 °C	Post-thaw	Bafra	Improved sperm CASA parameters, viability, PMI, having no effect on oxidation status and PRDX5 gene expression with reduced DNA damage and morphological abnormalities.	[145]
Cysteamine loaded on selenium-NPs (Cys-SeNps)	1 µg/mL	−196 °C	Post-thaw	Sanjabi	Improved sperm CASA parameters, viability, membrane/DNA integrity, SOD activity with reduced MDA content and morphological abnormalities	[70]
Zinc oxide nanoparticles	1 µg/mL	4 °C	72 h	N/A	Positive effect on sperm motility indexes, viability, membrane/DNA integrity, SOD activity and TAC with reduced MDA content and morphological abnormalities	[146]
Zinc oxide nanoparticles	10–200 µg/mL	−196 °C	Post-thaw	Santa Ines	No significant effect on sperm CASA parameters, PMI and ACI; however, significant improvement in sperm MMP.	[147]
CuO-NPs doped with ZnO-NPs & coated with quercetin	5 µg/mL	−196 °C	Post-thaw	Sanjabi	Improved sperm motility, viability, membrane/DNA integrity with significant decline in MDA content and morphological abnormalities.	[148]

Note: CASA: Computer-assisted sperm analysis; TAC: Total antioxidant contents; PMI: Plasma membrane integrity; SOD: Superoxide dismutase; DNA: Deoxyribonucleic acid; ACI: Acrosomal integrity; PRDX5: Peroxiredoxin-5; ROS: Reactive oxygen species; MMP: Mitochondrial membrane potential; MDA: Malondialdehyde.

## Data Availability

No new data were created or analyzed in this study. Data sharing is not applicable to this article.
